# Deciphering Allergen Peptides for Dermatological and Cosmetic Applications with Explainable Artificial Intelligence

**DOI:** 10.1021/acs.jproteome.6c00268

**Published:** 2026-06-25

**Authors:** Marina Geisiely Damaso, André Silva Pimentel

**Affiliations:** Departamento de Química, 28099 Pontifícia Universidade Católica do Rio de Janeiro, Rio de Janeiro,22453-900, Brazil

**Keywords:** allergen peptides, allergy, cosmetics, dermatology, explainable machine learning.

## Abstract

This study explores the potential of explainable artificial intelligence to advance our understanding of allergen peptides in the context of dermatology and cosmetics. We present a hybrid deep learning framework that integrates Temporal Convolutional Networks (TCN) and stacked Long Short-Term Memory (LSTM) architectures, enhanced with Evolutionary Scale Modeling (ESM) embeddings, to decode allergenic motifs embedded in peptide sequences. The ESM embeddings allow the model to capture both the evolutionary context and structural nuances of amino acids, enabling accurate classification of allergenic potential. Beyond classification, the framework emphasizes interpretability using state-of-the-art explainability tools such as Anchor, LIME, and SHAP. Anchor identifies minimal and decisive motifs responsible for allergenic activity, while LIME and SHAP provide a distributed importance map of *k-mers*, highlighting synergistic contributions across the peptide sequence. These methods ensure that the model output is not only precise, but it may also be biologically meaningful, opening a path to rational peptide modification strategies. In dermatology and cosmetic science, this approach represents a transformative tool, providing a scalable and transparent means to screen peptide-based formulations for allergenic risks, facilitates the design of safer bioactive peptides for therapeutic or cosmetic use, and supports regulatory compliance by grounding computational predictions in mechanistic explanations. Ultimately, the study bridges cutting-edge machine learning with dermatology and cosmetics, fostering the development of innovative, safe, and patient-centered cosmetic products.

## Graphical abstract

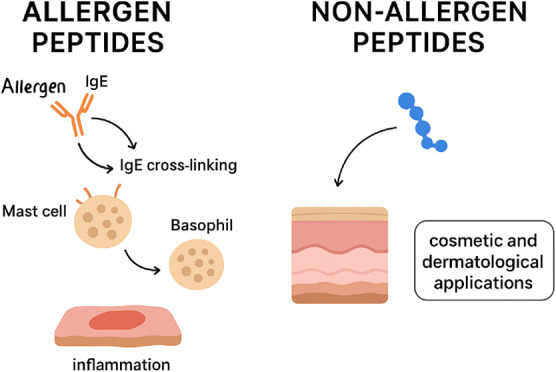

## Introduction

Allergen peptides are short amino acid sequences derived from larger allergen proteins that preserve key T-cell epitopes, reducing their immunoglobulin E (IgE)-binding capacity.^1–10^ They are fragments of an allergen (like pollen or dust mites) used in immunotherapy to desensitize the immune system and prevent allergic reactions. Recognition occurs by the immune system (via antigen-presenting cells, T cells, and B cells) and can bind to IgE antibodies on mast cells and basophils.^3,4,11^ This leads to the release of histamine and other mediators, causing allergic symptoms such as rash, visible symptoms, swelling, or dermatitis. Traditional allergic reactions in dermatology are dominated by a Th2-skewed profile, with interleukin-4 (IL-4), IL-5, and IL-13 driving IgE production and eosinophilic inflammation.^3,4,12,13^ Antiallergic peptides are derived from other sources, usually natural or synthetic compounds that can act by inhibiting histamine release, blocking IgE binding, or modulating immune cell activation to reduce inflammation and neutralize allergen-induced responses. They can block IgE binding sites on allergens, preventing cross-linking and mast cell activation. Some antiallergic peptides act as immune modulators, shifting the immune response from a predominantly Th2 allergic pathway to a tolerogenic or Th1/Treg profile. As antiallergic therapeutics, they can be used in conjunction with other treatments, such as antihistamines. The main difference is that allergen peptides are the “cause” of the allergy treated by immunotherapy,^1–10^ while antiallergic peptides are more like a drug that acts on symptoms or the cascade of the allergic response.^14,15^ The distinction between allergen peptides and antiallergic peptides is subtle but fundamental, especially in research in dermatology, cosmetics, and immunotherapy. When allergen peptides are administered, they stimulate T cells, eventually leading to a “counter immune” response that reduces or prevents the production of IgE antibodies and other allergic mediators upon future exposure to the allergen.^1–10^ They are used as a form of immunotherapy (allergen-specific immunotherapy, AIT),^2–10,12,13,16^ in which the patient is vaccinated with the allergen peptides to develop tolerance.^13^ In peptide-based AIT,^1–10,12,13,16^ peptides with short T-cell epitopes are used in controlled doses to induce immunological tolerance^13^ without triggering full-blown allergic reactions. In practice, peptide-based AIT^1–10,12,13,16^ has demonstrated several advantages over conventional approaches: reduced risk of systemic reactions, shorter treatment cycles, and greater patient compliance. Importantly, this strategy is not limited to treatment but also extends to prophylactic cosmetic applications, where peptide formulations can help prevent sensitization to common allergens.

Mechanistically, allergen peptides work by reshaping the immune response. By interacting with T cell epitopes in a controlled manner, allergen peptides can attenuate this response through the induction of regulatory T cells (Tregs) and a shift toward immune tolerance.^1–10,12,13,16^ Unlike full-length allergens, which can trigger immediate hypersensitivity through mast cell degranulation, allergen peptides primarily target T-cell activation, thus promoting tolerance induction without eliciting strong IgE-mediated reactions.^3,4,11,13^ This property makes them highly attractive not only for clinical interventions in allergic skin diseases but also for the development of safer dermocosmetic products.

In dermatology and cosmetic science, antiallergen peptides^14^ are designed to inhibit the interaction of allergen proteins with IgE for use in antiallergen skin care formulations.^17–19^ Therapeutic antiallergen peptides^14,15^ are being explored for the prevention of atopic dermatitis or cosmetic allergies.^17–19^ Proteins from fragrances and preservatives release allergen peptides upon degradation of skin creams, and allergen peptides from natural sources (e.g., pollen or plant extracts) can contaminate formulations and sensitize the skin.^5^ In cosmetic science, peptides in general represent a promising strategy for modulating immune responses to allergens commonly encountered through skin exposure, such as fragrances, preservatives, and dyes.^12,13,16,18,19^

Recent advances in artificial intelligence (AI) are accelerating allergen peptide research, particularly in dermatology and cosmetics. Sequence-based predictive models can identify allergen motifs, evaluate cross-reactivity, and propose safer peptide analogues for immunotherapy.^20–38^ The integration of large-scale protein language models, such as Evolutionary Scale Modeling (ESM),^39^ enables the extraction of biologically meaningful embeddings that capture both global sequence semantics and local structural features relevant to allergenicity. Furthermore, explainable AI (XAI) approaches, including high-precision model-agnostic explanations (anchor explanation is a rule that sufficiently “anchors”),^40,41^ Local Interpretable Model-Agnostic Explanations (LIME),^42^ and SHapley Additive exPlanations (SHAP),^43^ now allow researchers to pinpoint sequence motifs and structural determinants associated with allergenic potential, thereby bridging the gap between computational predictions and immunological interpretation.^96^ In the field of dermatology and cosmetics, these computational advances provide a powerful complement to experimental immunology. For example, machine learning-driven motif discovery can accelerate the design of hypoallergenic cosmetic formulations, while explainability methods help ensure regulatory transparency in safety assessments. At the same time, AI-guided peptide engineering opens avenues for personalized AIT approaches^1,2,6,12,16,30^ tailored to individual sensitization profiles. Thus, allergen peptides stand at the intersection of immunology, dermatology, cosmetic safety, and computational biology, forming the basis for innovative therapeutic and preventive strategies.

The central aim of this study is to develop and validate an explainable machine learning framework capable of classifying allergen peptides, identifying and decoding sequence motifs in allergen peptides that are directly associated with allergenic responses in dermatology and cosmetics. We sought to recognize both local patterns and long-range dependency modeling in peptide sequences by integrating Evolutionary Scale Modeling (ESM),^31,39^ which captures contextual and evolutionary information from protein sequences using a hybrid deep learning architecture combining Temporal Convolutional Networks (TCN) and stacked Long Short-Term Memory (LSTM) layers.^44^ This dual modeling strategy enhances the ability to detect subtle structural and sequential features that underlie allergenicity while maintaining interpretability through explainable AI methods such as Anchor,^40,41^ LIME,^42^ and SHAP.^43^ In doing so, the study aims not only to achieve high predictive performance but also to uncover biologically meaningful motifs and mechanistic insights, thereby advancing the scientific understanding of peptide-driven allergic processes and supporting the rational design of safer cosmetic formulations and peptide-based therapeutic interventions.

## Methodology

The foundation of any robust machine learning model lies in a carefully curated and balanced data set. In this study, allergen peptide data were systematically collected from literature^45^ and preprocessed to ensure both biological relevance and computational feasibility. The initial data set consisted of two primary sources: a curated library of 2462 experimentally validated allergen peptides, annotated as Allergen = 1, and a complementary data set of 333 nonallergen peptides derived from cosmetic and dermatological applications, annotated as non-Allergen = 0.^45^ Negative samples consisted of peptides reported in cosmetic and dermatological formulations with no documented evidence of allergenic activity in literature.^45^ None peptide was annotated as ambiguous, immunogenic, or lacking experimental characterization. To minimize hidden label contamination, duplicate sequences and highly similar peptides overlapping with the allergen class were removed prior to downstream analysis. These two sources were integrated into a single unified data set with 2795 peptides to enable a binary classification task capable of distinguishing allergen from nonallergen peptide sequences.

To address the inherent limitations of biological sequence data, including variability in sample size and class imbalance, a multistep augmentation and filtering strategy was applied. The sequence diversity was increased to improve model generalization by an intraclass sequence augmentation strategy. Pairs of peptides belonging to the same class were concatenated to generate synthetic hybrid sequences while preserving class-consistent immunological properties. First, a sequence-level mix up augmentation was performed within each class, a combination of all 2462 allergen peptides and all 333 nonallergen peptides. This procedure generated approximately 6.06 million allergen-derived combinations and 110,889 nonallergen-derived combinations prior to downstream filtering. Unlike classical numerical mix up, this strategy concatenated pairs of peptide sequences from the same label, generating synthetic hybrids that retained class-consistent immunological properties. The labels preserved biologically meaningful representations, ensuring that the augmented sequences have the same class of original sequences. This augmentation increased sample diversity and allowed the model to generalize across subtle sequence variations that might otherwise be underrepresented in the training data.

Following augmentation, sequence length constraints were applied to exclude peptides either too short (<7 amino acids) to drop peptides with low semantic complexity and encode relevant T-cell epitopes or too long (>40 amino acids) to preserve the structural homology of short allergen motifs. This filtering step ensured that only biologically plausible peptide candidates were retained for downstream analysis. The resulting data set was then rigorously cleaned to remove duplicate entries, reducing redundancy and preventing model overfitting to repeated sequences. Then, we sorted 20,000 peptides for each class, totalizing 40,000 peptides with reduced redundancy to avoid overfitting. From our experience, the data set size is suitable for training and validation using for advanced deep learning strategies. Because the original data set exhibited class imbalance, with allergen peptides substantially outnumbering nonallergen peptides, balancing procedures were implemented after augmentation. Initially, the minority class (allergens) and majority class (nonallergens) were separated, and the majority class was undersampled to match the size of the minority class. This undersampling step mitigated the bias toward predicting the dominant class, which is a frequent challenge in immunological data sets where nonallergens are more abundant. To further enhance fairness, both classes were randomly shuffled and resampled to a fixed number of samples per class (*n* = 20,000), ensuring equal representation of allergen and nonallergen sequences.

The final data set, therefore, represented a biologically filtered, synthetically augmented, and statistically balanced corpus of peptide sequences. This data preparation strategy allowed the downstream machine learning model not only to learn discriminative features between allergen and nonallergen peptides but also to remain resilient against overfitting, class bias, and data sparsity. By integrating this inspired augmentation, sequence-length filtering, duplicate removal, and balanced undersampling, the pipeline avoided data leakage, maximized both biological interpretability, and computational robustness, thereby providing a solid foundation for embedding extraction and model training. Then, we adopted a stratified split that preserves the positive/negative ratio. For model selection and generalization assessment, it was not necessary to perform k-fold cross validation or nested cross-validation. To prevent information leakage between subsets, redundancy filtering was performed prior to train/validation/test partitioning. Sequences sharing greater than 90% pairwise identity (see Table S1 since 80, 90, 95, and 99% pairwise identities were detected by using Jaccard similarity in high similarity pairs of 6-*mer* motifs) were removed from validation/test subsets, ensuring that highly similar peptides did not appear simultaneously across training, validation, and testing data sets. The final data set was divided into 80% training, 20% validation, and two independent testing sequences were previously taken before training: (1) 20 peptide sequences taken from original data set, and (2) 40 peptide fragments from real allergen proteins, which were split into peptide sequences. The step-by-step data set construction pipeline including the steps for redundancy filtering, train/validation/test separation, and for the prevention of overlap between subsets is presented in Figure 1.

**Figure 1:**
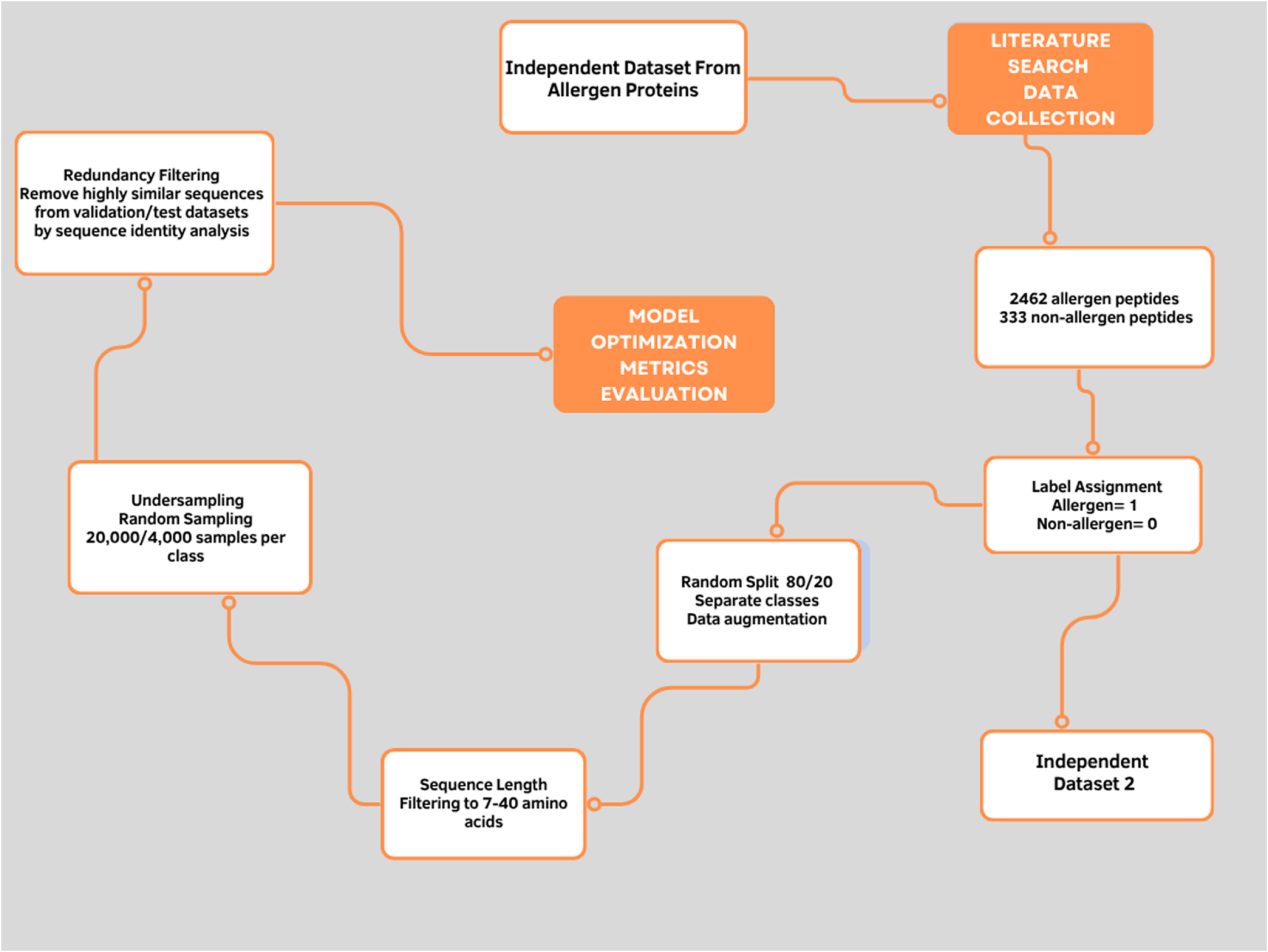
Step-by-step data set construction pipeline including the steps for redundancy filtering, train/validation/test separation, and for the prevention of overlap between subsets.

To enable efficient processing of peptide sequences while preserving both biochemical semantics and sequential organization, we employed a multimodal sequence representation strategy combining handcrafted one-hot encodings with contextual embeddings generated by a pretrained protein language model. This dual-representation framework was designed to capture complementary aspects of peptide information, including explicit residue identity, local compositional motifs, evolutionary constraints, and contextual biochemical dependencies associated with allergenicity. Initially, raw peptide sequences were transformed into one-hot encoded matrices using the 20 standard amino acids as the encoding vocabulary. In this representation, each amino acid position was mapped to a binary vector in which a single element corresponding to the residue identity was assigned a value of 1 while all remaining positions were set to 0. This encoding preserves exact positional residue information without introducing prior assumptions regarding physicochemical similarity or evolutionary relationships. One-hot representations are particularly useful for convolutional architectures because they allow the model to directly learn local compositional patterns and residue-level motifs from primary sequence data. The resulting matrices therefore provided the input representation for the Temporal Convolutional Network (TCN) pathway.

Because peptide sequences exhibited variable lengths, padding procedures were required to ensure consistent tensor dimensions across batches during training. A maximum sequence length of 100 residues was adopted, which exceeds the filtered peptide length range and avoids truncation of biologically relevant information. Sequences shorter than this threshold were padded using zero vectors applied to both left and right margins, thereby preserving central sequence information while maintaining fixed-dimensional input structures compatible with convolutional processing. Importantly, sequences longer than the defined threshold were not artificially truncated, preventing the loss of potentially relevant allergenic motifs or contextual sequence information. This strategy ensured computational consistency while minimizing distortion of the biological signal embedded within peptide sequences.

To further improve model robustness and reduce sensitivity to positional artifacts within the data set, reverse-sequence augmentation was additionally implemented during preprocessing. In this procedure, peptide sequences were reversed while preserving residue composition, generating alternative sequence orientations that encourage the network to learn generalized contextual relationships rather than overfitting to fixed positional biases. This augmentation strategy was particularly important for improving the stability of sequential feature extraction in the TCN pathway and for enhancing the capacity of the model to recognize distributed motif patterns independently of rigid sequence orientation.

In parallel with one-hot encoding, peptide sequences were also processed using ESM2-t6–8M-UR50D protein language model,^39^ a transformer-based protein language model pretrained on large-scale protein sequence databases. Unlike handcrafted representations, ESM embeddings provide dense contextual representations learned through self-supervised training on evolutionary sequence distributions. For each peptide, embeddings were extracted from the sixth hidden layer of the transformer architecture, which provides an effective balance between low-level structural sensitivity and higher-level semantic abstraction. Special beginning-of-sequence (BOS) and end-of-sequence (EOS) tokens were removed prior to aggregation to avoid introducing artificial contextual bias. Subsequently, mean pooling across sequence positions was applied to generate a fixed-dimensional embedding vector of size 320 for each peptide sequence.

These contextual embeddings encode evolutionary, biochemical, and structural information that cannot be directly represented through sparse one-hot encodings alone. In particular, ESM embeddings capture residue codependencies, contextual amino acid semantics, and long-range sequence relationships learned from large-scale protein models. Consequently, the integration of one-hot sequence representations with transformer-derived embeddings enabled the model to simultaneously exploit explicit local sequence information and high-level contextual biochemical knowledge. This multimodal encoding strategy formed the basis for the downstream hybrid architecture with a Temporal Convolutional Network (TCN) stacked and Layer-Normalized Long Short-Term Memory (LSTM) layers.^44^ This hybrid architecture was motivated by the need to simultaneously capture global evolutionary constraints, local sequential dependencies, and long-range contextual interactions that drive T-cell recognition of allergen peptides.

The encodings were fed into a TCN network, designed with stacked dilated causal convolutional blocks. Each temporal block employed gated convolutions with dilation factors increasing exponentially across layers, enabling efficient modeling of long-range dependencies. Residual connections, batch normalization, and GELU nonlinearities were incorporated to stabilize training and mitigate gradient degradation and loss oscillation in the validation. Dropout layers within each block (0.2) acted as regularizers, reducing the risk of overfitting. Global average pooling was then applied to summarize the TCN outputs into a compact feature vector. This vector captures global evolutionary regularities and smooths local noise.

The outputs from both the ESM embeddings and the TCN pathway were combined using an adaptive feature fusion module. This module projected the two feature spaces into a common latent dimension via fully connected layers followed by layer normalization and GELU activations. A gating mechanism, parametrized by a sigmoid function, adaptively weighted the contribution of each modality, effectively allowing the model to privilege evolutionary embeddings, local sequence features, or a balanced mixture depending on the input. This adaptive fusion strategy was critical for balancing complementary information sources, ensuring that sequence-specific motifs and evolutionary priors were jointly represented. The fused features were further processed by a Layer-Normalized LSTM consisting of three stacked layers with hidden units of size 128. This recurrent module was selected to capture temporal dependencies within the fused representation, simulating motif recurrence and long-range interactions that underlie allergenicity. Layer normalization was applied to hidden and cell states at each step, which has been shown to improve stability in deep recurrent architectures and reduce covariate shift. Dropout regularization (rate of 0.3) was included between layers to enhance generalization.

Finally, the sequence representations were fed into a classification head, implemented as a two-layer feedforward network. The first layer reduced the dimensionality of the LSTM output by half, followed by layer normalization, GELU activation, and dropout (0.4). The second layer produced logits corresponding to allergen versus nonallergen classes. Weight initialization (1 × 10^–4^ to 5 × 10^–4^) followed orthogonal initialization for recurrent components and Kaiming initialization for convolutional and linear layers, improving convergence speed and gradient flow. To further stabilize training, we employed label smoothing cross-entropy loss (ε ≈ 0.1), which redistributes a fraction of the probability mass across incorrect classes, mitigating overconfidence and improving calibration of predictions. Optimization was performed using Adam with gradient clipping and accumulation to prevent exploding gradients and stabilize updates. A hybrid learning rate schedule was adopted: an initial warmup phase linearly scaled the learning rate during the first ten epochs, followed by reducing learning rate on plateau (ReduceLROnPlateau) and cosine annealing to dynamically adapt learning rates based on validation loss. Early stopping was applied when no improvement was observed over five consecutive epochs, ensuring efficient convergence without overfitting.

This multimodal architecture thus integrates the evolutionary knowledge embedded in ESM, the local pattern detection of TCN, and the contextual memory of LSTMs into a unified explainable framework. By coupling high-capacity representation learning with transparent fusion and motif attribution methods, the model not only predicts peptide allergenicity but also provides mechanistic insights into the motifs and sequence contexts driving allergic responses.

To ensure a robust evaluation of the allergen peptide classification framework, we adopted a multilayered strategy that combined training dynamics analysis, statistical classification metrics, and error pattern visualization. This approach allowed us not only to quantify predictive accuracy but also to interpret model stability and diagnostic reliability across training and validation data sets. The training procedure was monitored for up to 100 epochs, with early stopping (patience of 10 epochs) to prevent overfitting and gradient clipping (threshold = 1.0) to mitigate gradient explosion in recurrent components of the architecture. Gradient accumulation over two steps was employed to balance stability with computational efficiency. The evolution of the training process was visualized through history plots that recorded the trajectory of the loss function and performance metrics across epochs. These plots provided insight into convergence behavior, optimization stability, and the onset of potential overfitting or underfitting phenomena. In particular, smooth convergence with minimal oscillations was considered indicative of effective learning, while divergence between training and validation curves suggested a loss of generalization capacity.

After training, the model was systematically evaluated on both the training and validation data sets using a set of complementary classification metrics. Accuracy quantified the overall proportion of correctly predicted peptides, while precision measured the proportion of predicted allergens that were true allergens, thus reflecting the model reliability in identifying clinically relevant peptides. Recall (sensitivity) captured the fraction of actual allergens that were correctly identified, thereby prioritizing the minimization of false negatives, which is critical in biomedical contexts where overlooking an allergen may have direct clinical consequences. The F1-score, as the harmonic mean of precision and recall, provided a balanced evaluation of the trade-off between these two measures. Beyond these conventional metrics, we also calculated Matthews Correlation Coefficient (MCC) and Cohen’s Kappa coefficient to assess model robustness in imbalanced scenarios. MCC, which incorporates true and false positives and negatives into a single statistic, is particularly valuable in binary peptide classification tasks where class distributions are not always balanced. Similarly, Cohen’s Kappa adjusts for agreement expected by chance, providing stricter criterion for assessing consistency between predicted and true labels. The combined use of these six metrics ensured that model performance was not only accurate but also statistically reliable and biologically meaningful.

To gain further insight into the distribution of classification errors, we generated confusion matrices for both the training and validation data sets. These heatmaps decomposed predictions into true positives, true negatives, false positives, and false negatives, thereby enabling direct inspection of systematic error patterns. For instance, a disproportionate number of false negatives would suggest that the model underestimates allergenic potential, whereas a prevalence of false positives would indicate an overly sensitive classifier. By visualizing these error structures side-by-side for training and validation data, we were able to assess not only internal consistency but also generalization across unseen data. This integrated evaluation strategy provided a comprehensive framework for assessing both the predictive accuracy and biological reliability of the allergen peptide classification model, encompassing training dynamics, statistical performance metrics, and error pattern visualization. The combination of conventional and robust statistical measures with visualization tools ensured that the model was validated not only in terms of raw accuracy but also in terms of interpretability and translational potential.

To elucidate the interpretability of our hybrid deep learning framework, we employed three complementary classes of model explainers: Anchor explanations,^40,41^ LIME,^42^ and SHAP.^43^ Each of these methods were adapted to the peptide allergenicity context by integrating *k-mer* based tokenization (*k* is a user-defined parameter, chosen 3 as an example) and model-specific prediction wrappers, thereby ensuring biological interpretability at the sequence motif level. Peptide sequences were tokenized into overlapping *k-mers* of length 3, 6, and 9, a representation that balances biochemical interpretability with the resolution necessary to capture immunologically relevant motifs. Both forward and reverse mapping were implemented, enabling the conversion of raw peptide sequences to *k-mer* inputs for the explainers, and vice versa. This allowed each explainer to interrogate the predictive model while preserving sequence fidelity.

First, we applied the Anchor method,^40,41^ which identifies sufficient conditions (“anchors”) in the form of *k-mers* whose presence alone is enough to reliably trigger an allergen prediction with high precision. To enable compatibility with the peptide space, we developed a lightweight tokenizer to treat *k-mers* as discrete tokens. Anchor explanations provide human-readable rules of the form “if these motifs are present, then the sequence is classified as allergen,” thereby supporting rule-based reasoning analogous to structural alerts in cheminformatics. Precision and coverage metrics were computed for each anchor, reflecting both the reliability of the motif and its generalizability across the data set.

Although LIME^42^ provides weighted feature importance, it does not guarantee stability of explanations across perturbations but complements the anchor explanation. We then employed LIME, which perturbs the input by randomly masking or altering *k-mers* and then fits a local surrogate model to approximate the decision boundary around the instance of interest. A custom wrapper was implemented so that perturbed *k-mer* sequences could be reassembled into valid peptides before being passed through the trained model. The explainer then quantified the relative influence of each *k-mer* on the probability of allergenicity, enabling the identification of short motifs that either drive or suppress allergen predictions. High-confidence allergen peptides (prediction probability >0.8) were prioritized for LIME analysis to reduce noise in local fidelity.

Finally, SHAP^43^ was applied to provide a unified, game-theoretic quantification of feature contributions. A deep explainer was constructed around our hybrid model, with embeddings from the ESM layer and one-hot encodings as joint inputs. Background sequences were sampled from the nonallergen class to provide a neutral reference distribution. SHAP values^43^ were then computed for selected allergen peptides, and residue-level contributions were aggregated to *k-mer* units. This allowed us to visualize positive or negative contributions of specific motifs toward allergenicity predictions, capturing both synergistic and antagonistic effects across residues. SHAP explanations were particularly useful for highlighting subtle distributed signals that might not appear in local surrogate models or anchor rules.

LIME,^42^ Anchor,^40,41^ and SHAP^43^ provide complementary perspectives: LIME identifies the most influential motifs in a local neighborhood, Anchor defines robust rule-like motif conditions, and SHAP quantifies global contribution distributions under a principled additive framework. By triangulating across these approaches, we achieved a more reliable interpretation of allergenicity-driving motifs, supporting both biological hypothesis generation and potential translation into immunoinformatics applications.

## Results and Discussions

Tables S1–S3 collectively provide a comprehensive, multilayered evaluation of data set similarity, diversity, redundancy for the predictive performance of the allergen classification framework. Table S1 demonstrates that no exact duplicates or CD-HIT cluster-level leakage are present across all data set configurations, confirming the absence of trivial redundancy, while motif-level (*k*-mer) and semantic similarities increase substantially in the augmented data set but are effectively minimized in the augmented data set with test data set splitting before augmentation, indicating successful mitigation of augmentation-induced leakage. Table S2 further characterizes the embedding space geometry, showing that allergen peptides (Class 1) exhibit consistently higher intraclass diversity in the original data set, while augmentation expands the nonallergen (Class 0) space and reduces interclass similarity, leading to a more distinct allergen manifold; importantly, permutation testing (*p* ≈ 0.000) and nonoverlapping bootstrap confidence intervals confirm that these diversity differences are statistically robust. Table S3 demonstrates that the multimodal ESM–TCN–LSTM model achieves high predictive performance across all data sets, with moderate generalization gaps in the original data set and near-perfect accuracy, MCC, and Cohen’s Kappa in both augmented scenarios; critically, the preservation of near-perfect validation performance in the leakage-controlled split confirms that model accuracy is not driven by redundancy but by learned biological signal, reflecting the integration of global evolutionary semantics and local sequence features, which is not possible to achieve using simple models despite their excellent accuracy performance (see Tables S4 and S5).

Figures S1–S4 provide complementary insight into model optimization dynamics and classification behavior. Figure S1 shows the training and validation loss trajectories across 35 epochs, where the original data set exhibits mild divergence indicative of limited generalization, while both augmented configurations display smooth and stable convergence with minimal oscillation, reflecting improved optimization stability and effective regularization. Figures S2–S4 further corroborate these observations through confusion matrices, where the original data set shows minor misclassification patterns, particularly reduced recall, whereas the augmented data set yields near-perfect classification but may reflect increased motif redundancy, and the augmented data set with test data set splitting before augmentation achieves near-perfect classification with balanced error distribution across both training and validation sets. These demonstrate that augmentation enhances model convergence and classification consistency, while test data set splitting before augmentation ensures that these gains are not confounded by information leakage.

Figures S5 and S6, based on UMAP and t-SNE dimensionality reduction, respectively, visualize the multimodal embedding space and reveal the underlying structure of peptide representations learned by the model. In the original data set, both UMAP and t-SNE projections show partially overlapping clusters, reflecting shared physicochemical and evolutionary constraints between allergen and nonallergen peptides. The augmented data set exhibits a denser and more expanded manifold, with improved separation between classes but increased local similarity due to motif reuse. In contrast, the augmented data set with test data set splitting before augmentation displays well-separated yet biologically coherent clusters, where allergen peptides occupy a distinct region of latent space while maintaining internal heterogeneity. The consistency between UMAP and t-SNE representations reinforces the robustness of the learned embedding geometry, indicating that the model captures meaningful structural and functional relationships rather than artifacts of dimensionality reduction. Collectively, these visualizations support the conclusion that allergenicity is encoded as a structured but heterogeneous region within protein sequence space, which can be effectively resolved through multimodal deep learning representations.

Tables S6–S9 show that the model performance for the testing data set with 20 samples (which was separated before splitting and contains 10 allergens and 10 nonallergens) after optimization of the deep learning model (TCN + LSTM) to classify allergen peptides using an Evolutionary Scale Modeling (ESM) language model (esm2_t6_8M_UR50D) to capture both global semantic and local structural features. It was found that the model predicts 10 out of 10 allergens and 8 out of 10 nonallergens, indicating one more time the model accuracy. Tables S6 and S8 also show comparison of Lime explanations of the RF (first row) and TCN/LSTM (second row) models using the original and augmented data sets, respectively. Tables S7 and S9 also show comparison of Lime explanations of the RF (first row) and TCN/LSTM (second row) models using the original and augmented data sets, respectively. These tables do confirm that the LIME and SHAP explanations of the TCN + LSTM model are different than the traditional models such as random forest. Table S10 summarize a comparison between our model and machine learning-based allergen peptide prediction models. In the Supporting Information, we also discuss our model with respect to recent related work highlighting what differentiates this approach from existing methods. A comprehensive ablation analysis was also performed using the original data set, systematically evaluating five distinct configurations: (i) the complete hybrid model, (ii) a model without the Temporal Convolutional Network (No TCN), (iii) a model without the LSTM module (No LSTM), (iv) a model without the feature fusion strategy (No Fusion), and (v) a baseline model relying exclusively on ESM embeddings without downstream sequential integration (ESM only). The results of these experiments are summarized in Table S11 and an interesting discussion is available in the Supporting Information.

The loss history presented in Figure 2 illustrates the evolution of both training and validation loss over the course of approximately 35 epochs. At the onset of training, the model exhibits relatively high loss values (≈0.33 for training and ≈0.28 for validation), which rapidly decrease within the first few epochs, reflecting the model swift adaptation to the underlying data distribution. Beyond this initial steep decline, the losses continue to decrease more gradually, stabilizing around 0.20 after approximately 20 epochs. Importantly, the training and validation curves follow one another closely across the entire training process, with no evidence of substantial divergence. This concordance suggests that the model generalizes well to unseen data, without exhibiting marked overfitting or underfitting. The minor oscillations observed in the validation curve are consistent with stochastic fluctuations inherent to the optimization process, particularly in models trained with mini-batch gradient descent. Overall, these results indicate that the learning dynamics are stable and that the model achieves a balanced fit, with both training and validation losses converging toward a low and nearly identical asymptote. This pattern strongly supports the robustness of the model architecture and its training regime.

**Figure 2:**
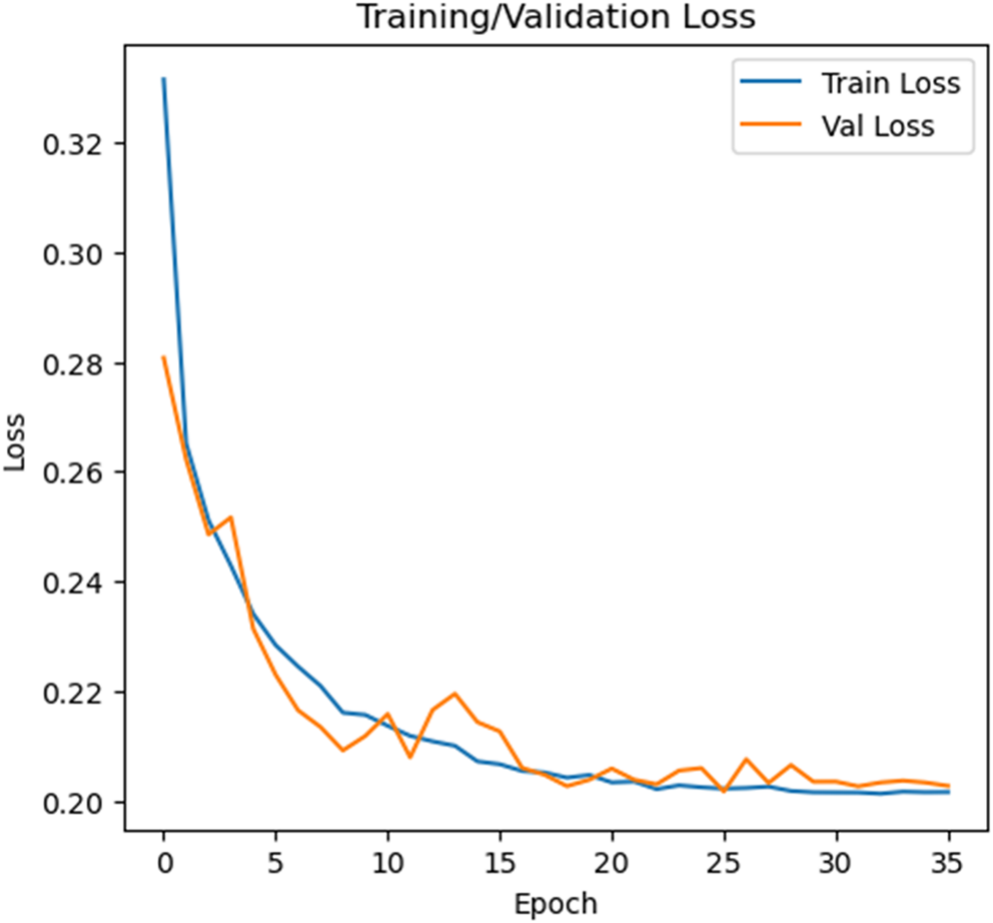
Training (blue line) and validation (orange line) losses for balanced augmented data set with 35 epochs during optimization of the deep learning model (TCN + LSTM) to respectively classify allergen peptides using an Evolutionary Scale Modeling (ESM) language model (esm2_t6_8M_UR50D) to capture both global semantic and local structural features.

The results summarized in Table 1 highlight the remarkable performance of the optimized deep learning architecture (TCN + LSTM enriched with ESM embeddings) in classifying allergen peptides. In the training data set, the model achieved perfect predictive capability, with all statistical measures (accuracy, precision, recall, F1 score, MCC, and Cohen’s kappa) reaching the maximum value of 1.000. This indicates that the model was able to fully capture the complex sequence patterns and semantic-structural dependencies embedded in the peptide data without any detectable misclassification. In the validation data set, the performance remained nearly optimal, with accuracy, F1 score, MCC, and Cohen’s kappa consistently exceeding 0.997, while precision reached 1.000 and recall slightly decreased to 0.997. These results demonstrate not only the ability of the model to generalize effectively to unseen data but also its robustness across multiple, independent evaluation metrics. The negligible discrepancy between training and validation performance suggests that the model avoids overfitting despite its high representational capacity, owing in part to the integration of the ESM language model, which provides biologically meaningful embeddings that balance global contextual understanding with local motif recognition. Taken together, the metrics confirm that the proposed approach delivers an exceptionally reliable classification framework, holding strong potential for translational applications in allergenicity prediction and peptide-based therapeutic design.

**Table 1:** Performance Statistical Metrics (Accuracy, Precision, Recall, F1 Score, Matthews Correlation Coefficient (MCC), and Cohen′s kappa) of Training and Validation Datasets after Optimization of the Deep Learning Model (TCN + LSTM) to Classify Allergen Peptides Using an Evolutionary Scale Modeling (ESM) Language Model (esm2_t6_8M_UR50D) to Capture Both Global Semantic and Local Structural Features

data set	accuracy	precision	recall	F1 score	MCC	Cohen′s Kappa
Training	1.000	1.000	1.000	1.000	1.000	1.000
Validation	0.998	1.000	0.997	0.998	0.997	0.997

The confusion matrices for the training and validation data sets in Figure 3 provide a detailed visualization of the classification performance of the optimized TCN + LSTM model enriched with ESM embeddings for allergen peptide prediction. In the training set, the model demonstrates near-perfect discrimination, with only three misclassifications out of over 32,000 samples (one false positive and two false negatives). This exceptionally low error rate underscores the model ability to capture the intricate sequence dependencies and biochemical patterns characteristic of allergenicity. In the validation set, the performance remains comparably robust, with 13 misclassifications across nearly 8000 samples (one false positive and 12 false negatives). Notably, most errors in both sets are attributable to false negatives, suggesting that while the model is highly sensitive and specific overall, a minimal fraction of allergen peptides may escape detection. The balanced diagonal dominance observed in both matrices confirms the stability and generalizability of the model across data sets, with error rates that are statistically negligible given the sample sizes. Taken together, these findings highlight that the integration of global semantic and local structural features through ESM embeddings enables the architecture to achieve an extraordinary level of precision and reliability, effectively bridging the gap between training optimization and real-world generalization in peptide classification tasks.

**Figure 3:**
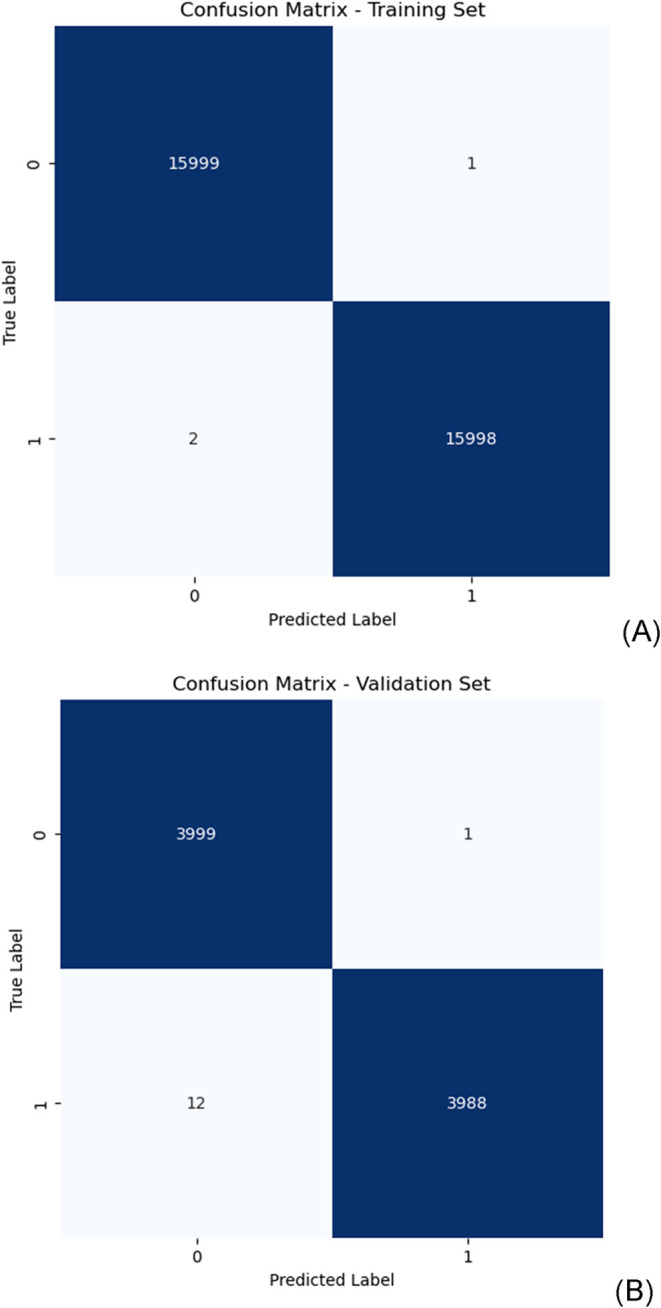
Confusion matrices of training (left panels) and validation (right panels) data sets after 35 epoch optimizations of the deep learning model (TCN + LSTM) to respectively classify allergen peptides using an Evolutionary Scale Modeling (ESM) language model (esm2_t6_8M_UR50D) to capture both global semantic and local structural features.

The interpretability provided by Anchor,^40,41^ LIME,^42^ and SHAP^43^ in Figure 4 collectively highlight the central role of specific proline- and glutamine-rich motifs in driving the deep learning model predictions for allergenicity. The Anchor method in Figure 4A identifies a deterministic set of 3-*mers* (e.g., **PAQ**, **QLQ**, **AQL**, **IQP**, **QDP**, **FPQ**, and **PQQ**) that, when present together, consistently lead the model to predict an allergen label with precision of 98.8% and full coverage. This deterministic rule-based explanation underscores the model reliance on well-defined motif patterns, many of which correspond to well-established immunogenic epitopes resistant to proteolytic cleavage. Except for the 3-*mers* **QDP**, these motifs are commonly found in gliadin-like proteins and other allergen peptides,^46–52^ providing biological plausibility to the computationally extracted features. In fact, the 3-*mers* motifs **FPQ** and **PQQ** are respectively found 7 and 19 times in the wheat protein, γ-gliadin, and found 34 and 68 times in seed storage wheat protein, ω-5 gliadin. Also, the 3-*mers* motif **FPQ** is also found in the wheat protein, profilin.^46–52^

**Figure 4:**
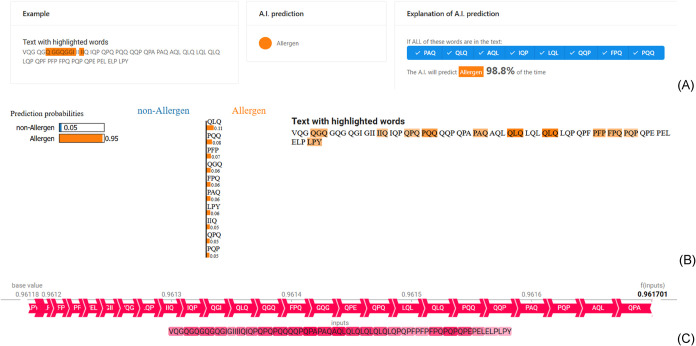
(A) Anchor, (B) LIME, and (C) SHAP explanations of the deep learning model (TCN + LSTM) with an Evolutionary Scale Modeling (ESM) language model (esm2_t6_8M_UR50D) to capture both global semantic and local structural features (3-*mer* motifs) important to interpret allergen peptides (sequence VQGQGI**IQPQQPAQLQLQ**P**FPQ**PELPY as example with the anchor PAQ + QLQ + AQL + IQP + LQL + QQP + FPQ + PQQ).

The LIME analysis^42^ offers a complementary, probabilistic perspective by ranking the local contributions of different 3-*mer* motifs to the allergen classification as presented in Figure 4B. Among these, γ-gliadin motifs^46–52^ such as **QLQ**, **PQQ**, **PFP**, **QGQ**, **FPQ**, and **PAQ** emerged as the most influential, with their weighted contributions strongly favoring the allergen class. In addition to the results found in Anchor explanations, the 2*-mers* motifs **PFP** and **QGQ** are found in γ-gliadin.^46–52^ Importantly, LIME highlights that these motifs exert additive effects, and their presence in combination amplifies the probability of allergenicity prediction. This ranking aligns closely with immunological knowledge that repetitive P- and Q-rich motifs (e.g., proline (P) and glutamine (Q)) can form structural clusters recognized by immune receptors,^46–52^ suggesting that the model is not only fitting to statistical patterns but also capturing biologically relevant determinants of allergenic responses.

The SHAP analysis^43^ further validates these findings by quantifying the global contribution of each 3-*mers* motif across the sequence in Figure 4C. It demonstrates consistent positive shifts in the prediction score when γ-gliadin motifs^46–52^ such as **QLQ**, **PQQ**, **FPQ**, and **PAQ** are present. The monotonic progression in SHAP contributions indicates that these motifs exert strong and cumulative effects, reinforcing the allergen classification in a transparent and interpretable manner. Taken together, the integration of Anchor,^40,41^ LIME,^42^ and SHAP^43^ highlights the synergy between rule-based anchors, probabilistic motif importance, and cumulative contribution dynamics. This interpretability framework not only confirms the reliability of the TCN + LSTM model but also provides molecular-level insights into allergenicity, bridging computational peptide modeling with immunological mechanisms.

In Figure 5A, the Anchor method identifies specific 6-*mers* motifs (namely **QQPYPQ**, **QPYPQQ**, and **PYPQQP**) as highly predictive of allergenicity, with a precision of 96.4% and full coverage, indicating that the model reliably recognizes these overlapped γ-gliadin motifs as sufficient conditions for classifying peptides as allergens. These γ-gliadin motifs, recurrently highlighted in the input sequence, reflect short contiguous amino acid patterns that are likely to interact with IgE or T-cell receptors,^46–52^ emphasizing the model capacity to capture functionally relevant local sequence features.

**Figure 5:**
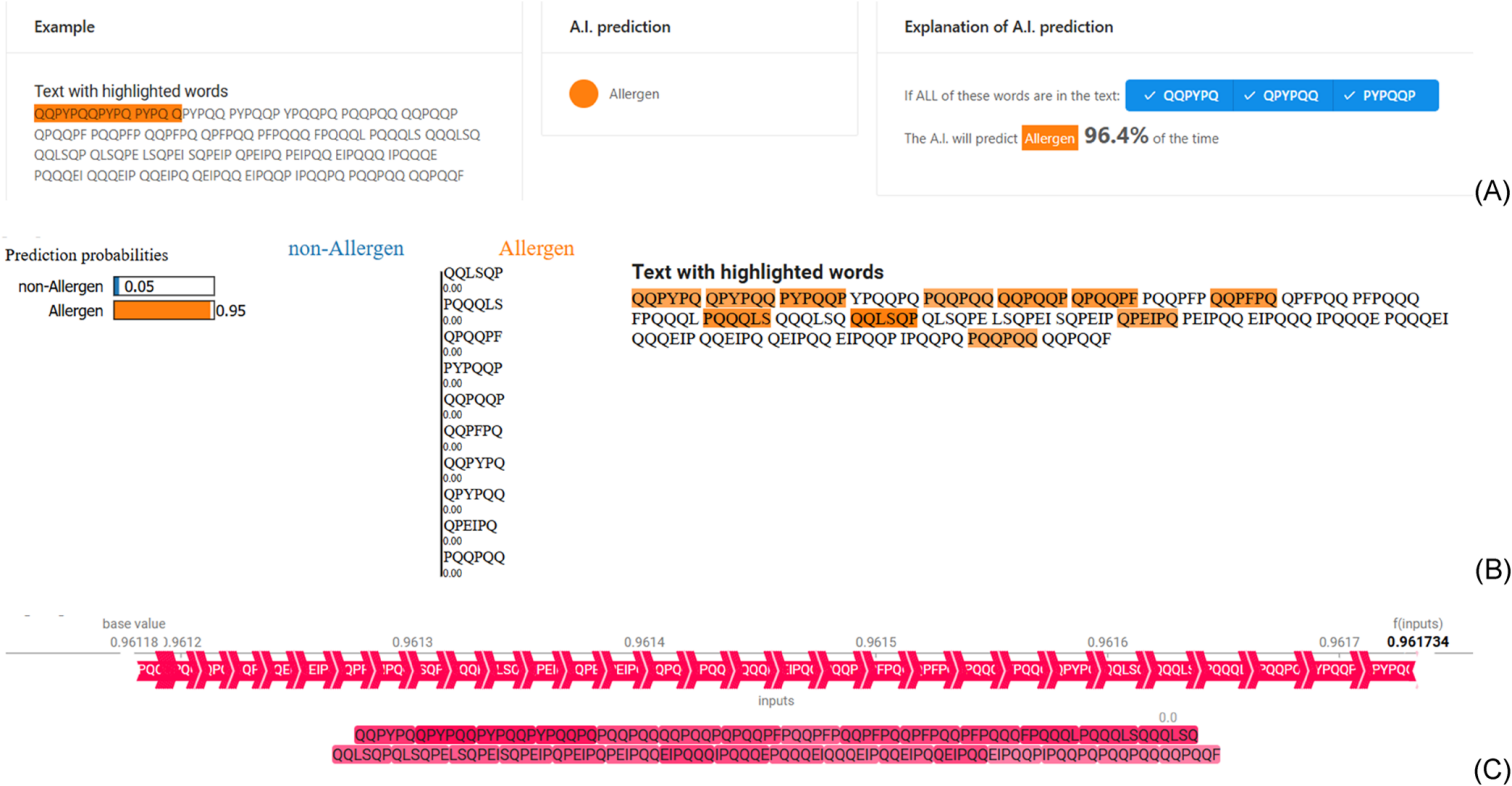
(A) Anchor, (B) LIME, and (C) SHAP explanations of the deep learning model (TCN + LSTM) with an Evolutionary Scale Modeling (ESM) language model (esm2_t6_8M_UR50D) to capture both global semantic and local structural features (6-*mer* motifs) important to interpret allergen peptides (sequence **QQPYPQQP**QQPFPQQQLSQPEIPQQQEIPQQPQQF as example with the anchor QQPYPQ + QPYPQQ + PYPQQP).

Complementing the Anchor results, the LIME analysis^42^ in Figure 5B quantitatively ranks 6-*mers* motifs based on their contribution to the model decision, highlighting sequences such as **QQLSQP**, **PQQQLS**, and **QPQQPF** as top contributors. From these three motifs, the **QPQQPF** 6-*mers* motif is the only one found in γ-gliadin.^46–52^ This demonstrates the model sensitivity not only to previously known allergenic motifs,^46–52^ but also to surrounding sequence context, which may modulate immunogenicity. The consistency between Q- and P-enriched motifs identified in Anchor^40,41^ and LIME^42^ suggests that the model internal representations, derived from the ESM2_t6_8M_UR50D language model embeddings, effectively integrate both local 6-*mers* motifs information and broader evolutionary and structural patterns, allowing for nuanced discrimination between allergen and nonallergen peptides.

Figure 5C presents the SHAP analysis^43^ of 6-*mers* motifs providing a positional, base-level explanation of the model prediction, and confirming that the highlighted residues consistently increase the probability of allergen classification. The attribution map indicates that contributions from motifs like **QPYPQQ** and **QPEIPQ** are positive across the sequence, underscoring the additive nature of motif-based features in driving allergenicity predictions. From these two motifs, the **QPQQPF** 6-*mers* motif is the only one found in γ-gliadin.^46–52^ Collectively, these results demonstrate that the deep learning model predicts biologically significant 6*-mer* motifs, validated through multiple explainable AI frameworks, and that the integration of ESM-derived embeddings with TCN and LSTM layers allows for precise and interpretable prediction of peptide allergenicity, aligning well with known immunological mechanisms.^46–52^

The Anchor explanations^40,41^ reveal in Figure 6A that the model assigns particularly strong predictive weight to the 9*-mers* motif **QQPYPQQPQ**, achieving a precision of 98.3% in classifying peptides as allergens when this subsequence is present. This indicates that the model has identified specific conserved 9*-mers* fragments that are highly indicative of allergenicity, reflecting biologically meaningful sequence patterns associated with immune recognition. In fact, **QQPYPQQPQ** is found in γ-gliadin.^46–52^ However, the coverage of the 9*-mers* motif was zero, and we also tried to get coverage of 100% with 8*-mers*, but this was not achievable. Anchors thus provide a rule-based interpretive scaffold, where the presence of certain subsequences (3-*mers* and 6-*mers*, but not 8- and 9*-mers*) suffices to trigger an allergenic classification with high confidence.

**Figure 6:**
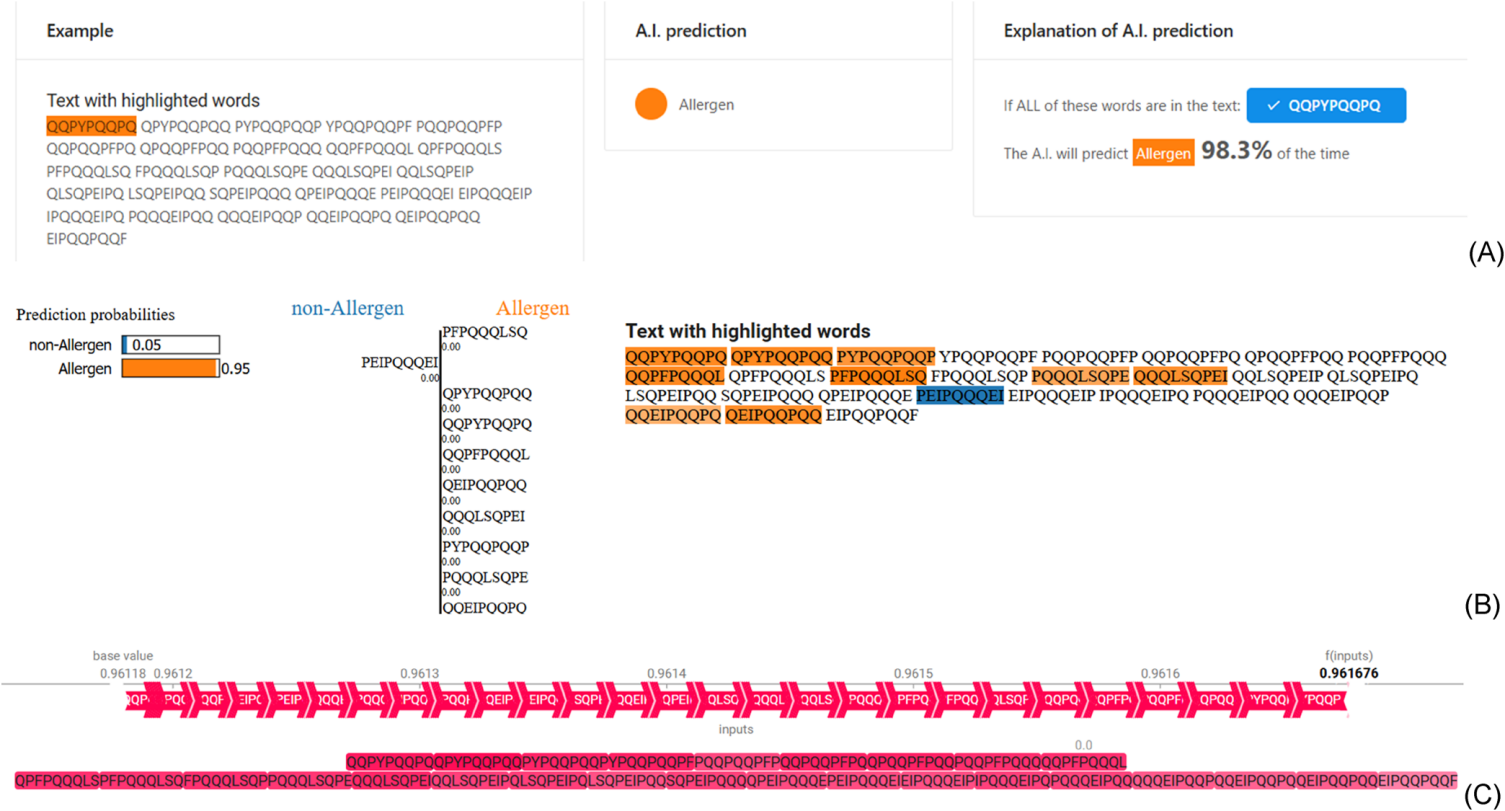
(A) Anchor, (B) LIME, and (C) SHAP explanations of the deep learning model (TCN + LSTM) with an Evolutionary Scale Modeling (ESM) language model (esm2_t6_8M_UR50D) to capture both global semantic and local structural features (9*-mer* motifs) important to interpret allergen peptides (sequence **QQPYPQQP**QQPFPQQQLSQPEIPQQQEIPQQPQQF as example with the anchor QQPYPQ + QPYPQQ + PYPQQP).

The LIME explanations^42^ in Figure 6B extend this understanding by highlighting influential motifs within the input sequence. Positive contributing 9-*mers* motifs, such as **QQPYPQQPQ**, **PFPQQQLSQ**, and **QQQLSQPEI**, align with the findings from Anchor method, suggesting their role as allergenic determinants. From these three motifs, the **QQPYPQQPQ** 9*-mers* motif is the only one found in γ-gliadin.^46–52^ Interestingly, the motif **PEIPQQQEI** was assigned to make a negative contribution, suggesting it counteracts allergenicity predictions when present, thereby revealing potential sequence contexts that reduce allergenic likelihood. The local perturbation-based LIME analysis underscores that allergenicity classification is not solely driven by the presence of one motif but by a weighted interplay of multiple overlapping 9*-mers* motifs, consistent with immunological evidence that allergenic epitopes are influenced by sequence context and surrounding structural features.

Finally, the SHAP analysis^43^ in Figure 6C provides a more fine-grained and additive perspective on global feature contributions across the sequence. The visualization indicates a cumulative positive effect of 9*-mers* motifs such as **QQPYPQQPQ** and **QQQLSQPEI** toward allergenicity, in agreement with Anchor and LIME, while also quantifying the marginal contributions of individual subsequences in a global explanatory framework. Each contributing motif highlights the synergistic effect of multiple allergenic determinants along the peptide chain. From these two motifs, the **QQPYPQQPQ** 9*-mers* motif is the only one found in γ-gliadin.^46–52^ Taken together, these interpretability approaches triangulate on consistent findings about the influence of Q- and P-enriched motifs: the deep learning model not only learns immunologically plausible allergen-associated motifs but also differentiates between positive and negative modulating subsequences. This highlights the utility of combining TCN + LSTM with ESM embeddings for biologically grounded allergenicity prediction and demonstrates how interpretable AI can bridge black-box predictions with mechanistic insights into peptide immunogenicity.

## Implications

Table 2 illustrates the application of the XAI pipeline to allergen classification, in which real full-length allergen protein sequences were segmented into peptide sequences, which contains overlapping 6*-mers* motifs to allow efficient computational processing. Each segment was analyzed using LIME method, which identifies 6*-mers* motifs contributing either positively or negatively to allergenicity predictions. Yellow highlights mark overlapped 6*-mers* motifs with a positive influence, meaning they significantly drive the classification model toward predicting allergenicity, whereas turquoise highlights correspond to overlapped 6*-mers* motifs with a negative influence, decreasing the probability of allergen classification. Motifs without highlights are considered noninfluential in the prediction. This analysis demonstrates the utility of the XAI pipeline in decoding allergenicity predictions at a fine-grained resolution, pinpointing specific motifs that drive classification decisions. Highlighting these motifs allows researchers to interpret allergen model outputs biologically, linking positive contribution motifs to known allergenic determinants, while identifying segments with neutral or negative contributions. Such insights enhance transparency in allergen prediction and can guide rational peptide or protein engineering or epitope-focused studies by delineating immunogenic hotspots and tolerance-associated motifs. From what is known from experiments,^53^ some examples of allergen protein from Table 2 are described in the following:

(1) Profilins have allergenic motifs that are short sequential segments (≈6–12 amino acids) within loops bordering the actin-binding and poly-l-proline (PLP) faces. They are enriched in lysine/acidic residues, often contain glycine-rich turns for flexibility, and contrast with buried β-strand motifs that are usually noncontributory. Structural and immunological mapping shows these motifs carry the greatest allergenic weight, with homologous patches in cucumber/melon and grasses explaining cross-reactivity. Pathogenic motifs combine B-cell 6–12-mers with adjacent HLA-II cores. pH-dependent conformational changes may modulate motif exposure.^54–60^

(2) The motifs of **α-amylase/trypsin inhibitors (AAIs/ATIs)** are 6–12 amino acid surface-exposed loops or helix-loop junctions, enriched in polar/charged residues and flanked by cysteines, sometimes adjacent to glycosylation sites. Validated epitopes (CM3/Tri a 30, 0.19/0.28 inhibitors) correlate with strong IgE binding and overlap innate immune–active regions. Buried β-strand motifs are generally noninfluential.^56–59^

(3) The **nsLTP1** motifs **(e.g., Pru p 3)** are in three dominant regions (∼11–25, ∼31–45, ∼71–80), forming solvent-exposed loops or helix termini stabilized by cysteines. They are 6–12 amino acid, charged/polar, often glycine-rich, and drive cross-reactivity with Ara h 9 and Jug r 3. Additional conformational epitopes occur near the lipid-binding pocket.^61–65^

(4) The **Agglutinin isolectin 1 (Wheat germ agglutinin) has the** IgE-binding motifs with 6–12 amino acid solvent-exposed peptides near carbohydrate-binding pockets and oligomer-interface loops, combining ligand-binding activity with multivalent surface exposure.^66–69^

(5) The motifs of **thioredoxins (Tri a 25, Zea m 25)** include the active-site CXXC sequence with flanking residues, and 6–12 amino acid surface-exposed loops or helix termini enriched in polar residues. Cross-reactivity with human thioredoxin derives from conserved motifs.^70–74^

(6) The Linear epitopes **of α-Purothionin (Tri a 37)** mapped to N- and C-terminal peptides, with minimal 6–12 amino acid motifs such as KSCCRS, CCRSTL, STLGRN, CNLGCR, and GCRSSV. These motifs are cysteine-adjacent, enriched in basic or acidic residues, and stable against digestion.^75–77^

(7) The candidate motifs of **MUL1** are 6–12 amino acid solvent-exposed loops or termini, particularly in the ring domain, though MUL1 lacks classical allergen features. Testing should focus on major histocompatibility complex (MHC)-II–binding cores within such regions.^78–81^

(8) The **PR60** motifs are likely cysteine-flanking loops, short disulfide-stabilized peptides, helix-loop termini, and charged/polar patches in flexible turns, like nsLTP motifs.^82–85^

(9) The **Elongation Factor-1 (EF-1α)** motifs are 6–12 amino acid solvent-exposed loops or termini, with relevance when divergent from human EF-1. Proteomic persistence and Human Leukocyte Antigen Class II (HLA-II) binding potential increase clinical importance.^86–90^

(10) **LMW glutenin (GluB3–23) has** two main motif classes: (1) repetitive Q/P-rich 6–12 amino acid stretches in the central domain, forming multivalent linear epitopes; and (2) cysteine-flanking surface loops in C-terminal domains, forming linear or disulfide-stabilized conformational epitopes.^91–93^

**Table 2:**
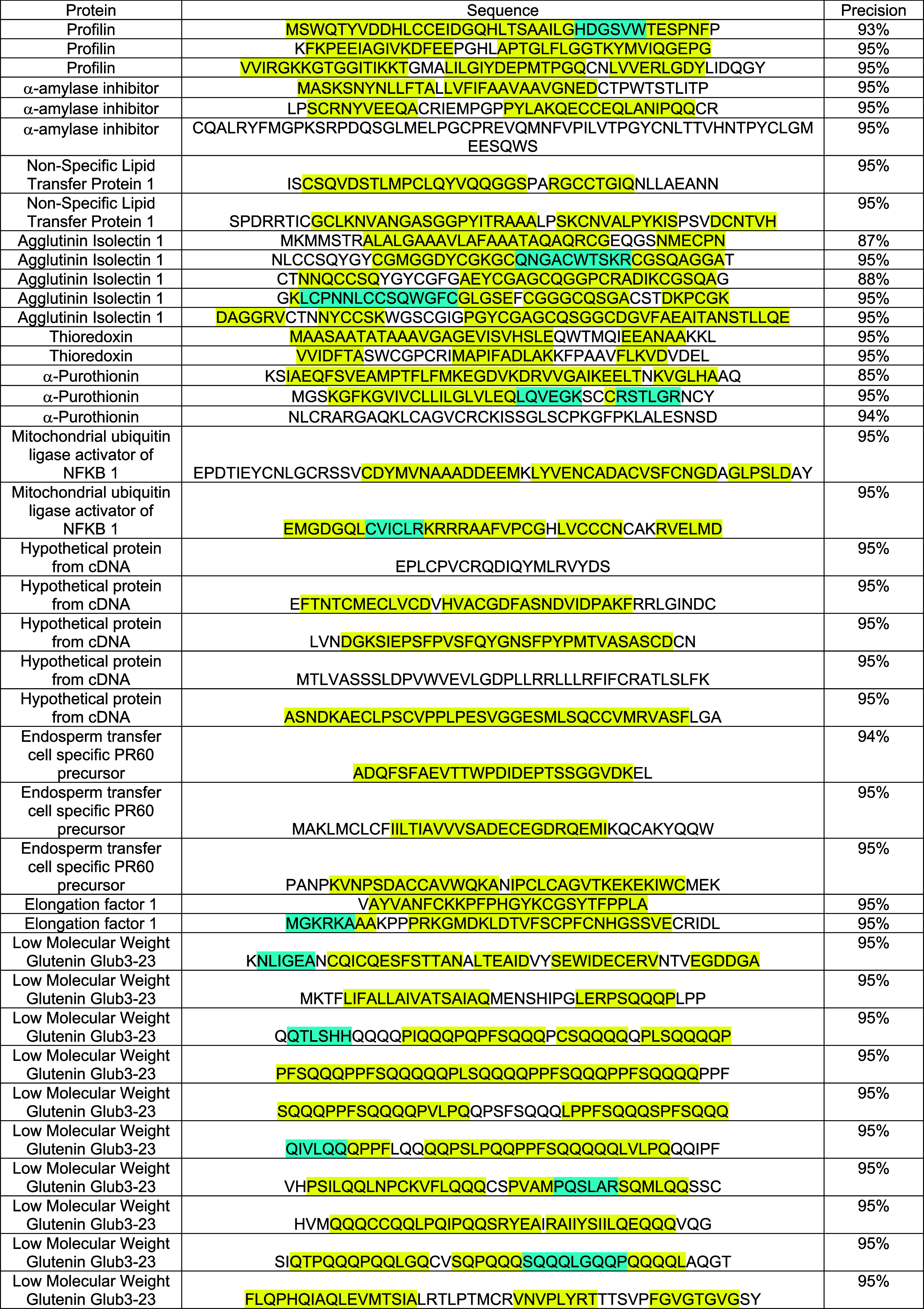
Application of the Explainable Artificial Intelligence (XAI) Pipeline in Real Allergen Proteins, Which Were Split into Peptide Sequences to Overcome the High Memory Demand and Computing Time to Apply the Pipeline^a^

a The yellow and turquoise highlights are overlapped 6*-mers* motifs found by LIME analysis that cause positive and negative allergen influence, respectively. The motifs without highlight are not influential. The classification model classifies the peptide sequence as allergen at the respective given precision. Note: The highlights only represent the appearance in the LIME analysis, they do not represent a colormap with a color scale.

Allergies caused by cosmetic ingredients represent a convergence of molecular immunology, dermatology, and consumer safety. Allergen peptide research shows that short proline- and glutamine-rich motifs, such those found in γ-gliadin for example^46–52^ are central to IgE-mediated sensitization and T-cell activation. Hydrolyzed wheat proteins (HWPs), widely used in cosmetics, are a prime example: in Japan, thousands developed wheat-dependent exercise-induced anaphylaxis and urticaria after exposure to HWP-containing soap. Protease-resistant gliadin-derived peptides can penetrate the skin barrier, retain IgE-binding capacity, and trigger degranulation in sensitized individuals, illustrating how repetitive motifs act as stable sensitizers.^94,95^ These motifs persist due to their conformational rigidity, high proline/glutamine content, and resistance to proteolysis, enabling them to carry overlapping IgE and 9*-mers* T-cell epitopes that bind HLA-DQ2/DQ8 and amplify Th2-driven responses. Surfactants in cosmetics further enhance dermal penetration, transforming routine products into systemic immunological risks. Detection tools, like the R5 monoclonal antibody, target **QQPFP**-like epitopes found in γ-gliadin,^46–52^ reinforcing their biological relevance and utility for allergen monitoring.

In summary, interpretable deep learning models trained on peptide ESM2 embeddings provide structural insight. Anchor analysis identifies deterministic motifs (e.g., **QQPYPQQPQ, QQPYPQ, QPYPQQ, PYPQQP**, **QLQ, QQP, PQQ**) as sufficient for allergen classification. LIME highlights additive effects of peptide motifs (e.g., **PFPQQQLSQ, QPYPQQPQQ, QQPYPQQPQ, QQLSQP, PQQQLS, QPQQPF, PQQPQQ, QLQ, PQQ, QPQ, PQP**) that may explain allergenicity. SHAP confirms cumulative positive contributions of motifs like **QQPYPQQPQ**, **QPYPQQPQQ, PYPQQPQQP, QQQLSQPEI, QPYPQQ, PYPQQP, YPQQPQ, PQP, QQP, PQQ**, mirroring immunological principles of overlapping epitopes as presented in Figures 4, 5, and 6 for the exemplified peptides that were also found in Table 2.

The integration of clinical evidence, immunological data, and explainable AI creates a robust framework for predicting and preventing cosmetic-induced allergies. Computational motif discovery aligns with real-world outbreaks, validating models as biologically grounded. By screening for high-risk sequences, these tools can reduce sensitization risks, guide safer peptide engineering, and inform regulatory oversight. Future cosmetic safety depends on systematically eliminating allergenic motifs, optimizing composition, and integrating predictive AI with rigorous biochemical validation. This combined approach offers mechanistic clarity, early allergen detection, and practical strategies for preventing sensitization, advancing skin care innovation without compromising consumer safety.

## Limitations

It is important to note some points and potential limitations of this research. Regarding the LIME and SHAP techniques, it is important to understand that they do demand unfeasible computational time and memory for large proteins. It is necessary to fragment the protein into parts of no more than about 40 amino acids to obtain explanations. In the Anchor technique, however, the protein must be fragmented into even smaller parts to generate explanations. Also, the models trained in this work considered only the mechanisms of action for which they were trained. We understand that different mechanisms of action exist within immunology, and there are still mechanisms under study for further elucidation. Therefore, we cannot claim to have used all possible existing mechanisms of action, as this may change depending on the site of action and route of administration.

Another important point is that the model is not trained to predict the action of an amino acid sequence that is hidden in a folded protein. Therefore, the model does only recognize an amino acid sequence that is on the protein surface. Unlike the experiment, which only considers amino acids that remain on the surface and are capable of interacting with the target, the data augmentation technique assumes that an allergen peptide (AAAAA) when mixed with another allergen peptide (BBBBB) forms a new allergen peptide (AAAAABBBBB). Similarly, a nonallergen peptide (CCCCC) when mixed with another nonallergen peptide (DDDDD) forms a new nonallergen peptide (CCCCCDDDDD). However, this assumption has not been experimentally verified. Finally, it is worth noting that no model is perfect or 100% effective; the limitations of computational models must be considered, even if minimal in some cases.

## Conclusions

The integration of Temporal Convolutional Networks (TCN) and Layer-Normalized LSTMs with evolutionary-scale embeddings (ESM2) demonstrates a powerful and interpretable framework for predicting peptide allergenicity. This hybrid model uses the representational strength of ESM embeddings in capturing evolutionary and structural context, while the TCN–LSTM architecture effectively encodes both local motif patterns and long-range sequence dependencies. As a result, the model not only achieves high predictive accuracy but also provides mechanistic insights into allergenic sequence determinants. The incorporation of explainable AI methods (Anchor, LIME, and SHAP) further enhances the interpretability of predictions by offering complementary perspectives. Anchor identifies minimal and sufficient motifs with high precision, representing deterministic rules of allergenicity. LIME highlights the probabilistic contributions of distributed *k-mers*, uncovering cooperative interactions between overlapping motifs. SHAP provides a global attribution framework, quantifying additive contributions of individual residues and distinguishing between motifs that enhance or mitigate allergenic potential. Together, these approaches triangulate consistent findings, reinforcing both the biological plausibility and computational reliability of the model decision-making. From a translational perspective, this interpretability framework holds significant promise for dermatology and cosmetic science. By systematically identifying and characterizing high-risk motifs, it enables the rational design of hydrolyzed peptides and protein fragments with reduced allergenicity, while also recognizing protective sequence contexts. Such a strategy embeds transparency and mechanistic clarity into predictive modeling, bridging computational peptide science with regulatory and clinical imperatives. Ultimately, this work provides a rigorous foundation for the development of next-generation dermatological and cosmetic products that are not only effective but also demonstrably safe for consumers.

## Supplementary Material



## Data Availability

Code, input data, and processing scripts for this paper are available at the GitHub repository (https://github.com/andresilvapimentel/Allergen-peptide-explainer). The data analysis scripts of this paper are also available in the interactive notebook Google Colab (for running jobs on an NVIDIA A100 80GB with 9.7 TFLOPS or NVIDIA A100 40GB with 9.7 TFLOPS GPUs) or Jupyter notebook (for running jobs locally on a PC I9 14900K, PL Z790 M 64 GB Fury with an NVIDIA RTX4090 24GB GPU).

## Notes

Data leakage analysis; detailed characterization of the peptide representation space; performance metrics for training and validation; evaluation of machine learning and deep learning models, and explanation models; comparison of machine learning and deep learning models; ablation studies; deep learning model performances (PDF)
