# Supplementary material for: Deciphering Allergen Peptides for Dermatological and Cosmetic Applications with Explainable Artificial Intelligence
Source: J Proteome Res. 2026 Jun 25;25(8):4212–28. doi: 10.1021/acs.jproteome.6c00268 (PMC13459551; doi:10.1021/acs.jproteome.6c00268)
Supplement: Supplementary file 1 [file pr6c00268_si_001.pdf]

Deciphering Allergen Peptides for Dermatological and Cosmetic Applications with Explainable Artificial Intelligence

Marina Geisiely Damaso[1] and André Silva Pimentel[1,*]

1. Departamento de Química, Pontifícia Universidade Católica do Rio de Janeiro, Rio de Janeiro, RJ 22453-900 Brazil

*Corresponding author: a_pimentel@puc-rio.br

Supporting Information

Table of contents:

Data leakage analysis	S-2
Detailed characterization of the peptide representation space	S-5
Performance metrics for training and validation	S-9
Evaluation of Machine Learning and Deep Learning Models, and Explanation models	S-15
Comparison of Machine Learning and Deep Learning Models	S-26
Ablation Studies	S-30
Deep Learning model performances	S-34

Data leakage analysis

The data leakage analysis presented in Table S1 provides a rigorous, multi-scale evaluation of redundancy between training and validation datasets across three experimental configurations: the original dataset (2236 training / 559 validation sequences), the fully augmented dataset (32000 training / 8000 validation sequences), and the augmented dataset with splitting of a small test dataset (20 samples, 10 allergens and 10 non-allergens). The analysis integrates sequence identity clustering (CD-HIT), motif-level similarity (*k-mer* Jaccard index with k = 6), and representation-level similarity derived from protein language model embeddings, thereby offering a comprehensive assessment of potential information leakage from both a sequence-centric and embedding-centric perspective.

At the most stringent level, no exact duplicates (0) or CD-HIT cluster leakage (0) were observed across all three dataset configurations with cutoffs 0.80, 0.90, 0.95, and 0.99. This indicates that neither identical sequences nor high-identity homologous clusters (as defined by CD-HIT) are shared between training and validation sets. From a statistical learning standpoint, this confirms that the datasets are not trivially overlapping at the primary sequence level, and that the model is not benefiting from memorization of identical or near-identical peptides. This observation is particularly important given the application of data augmentation, as it demonstrates that augmentation did not introduce explicit redundancy across splits. This implies that validation samples do not occupy identical or near-identical coordinates in sequence identity space relative to training samples.

However, differences emerge when examining motif-level and semantic similarity metrics. In the original dataset, *k-mer* leakage (100 pairs) and semantic leakage (538 pairs) are relatively modest, reflecting the limited size of the dataset and the inherent reuse of short peptide motifs. The presence of 1899 cross-nearest-neighbor relationships indicates that, even in the absence of identity-based redundancy, many pairs of validation sequences with cross-split k-nearest neighbors (k = 5) lie within the local neighborhoods of training peptides in the learned embedding space. This is consistent with the known structure of protein sequence space, where peptides sharing physicochemical or immunogenic

properties cluster together despite low global sequence identity. In contrast, the augmented dataset exhibits a substantial increase in similarity-based interactions using the Jaccard similarity of 0.8, with 361 *k-mer* leakage pairs, 7982 semantic leakage pairs, and 26789 cross-nearest-neighbor connections. After increasing the Jaccard similarity to 0.9, 0.95, and 0.99, the *k-mer* leakage was reduced to 82, 14, and 0 as shown in Table S1. The performance metrics in Table S3 were maintained after filtering these *k-mer* pairs. This amplification reflects the effect of augmentation strategies that generate sequences with preserved or recombined local motifs. While exact duplication is avoided, augmentation increases the density of the sequence manifold, leading to a higher probability that validation sequences share local subsequences or embedding proximity with training samples. From a geometric standpoint, this results in a denser occupation of latent space, where clusters corresponding to allergenic and non-allergenic patterns become more populated. Importantly, this does not necessarily indicate invalid leakage but rather highlights that augmentation enriches motif reuse and structural similarity, which may contribute to a slightly improved model performance through enhanced representation learning rather than simple memorization.

The most critical observation arises from the augmented dataset with test dataset splitting before augmentation, where leakage metrics are dramatically reduced to 0 *k-mer* leakage, 17 semantic leakage pairs, and 88 cross-nearest-neighbor relationships. This configuration effectively eliminates motif-level redundancy while preserving a minimal level of semantic proximity that is expected in any biologically realistic dataset. The near-complete removal of *k-mer* overlap demonstrates that augmentation-induced motif propagation can be mitigated through this appropriate dataset splitting strategy. The residual semantic and nearest-neighbor similarities likely reflect unavoidable biological convergence, where distinct peptides share higher-order structural or physicochemical properties captured by the embedding model. From a statistical perspective, this dataset configuration provides the strongest evidence of independence between training and validation sets, ensuring that model evaluation reflects true generalization rather than information leakage.

Table S1. Data leakage summary to the validation sequences using the original dataset (2236 sequences for training and 559 sequences for validation), the augmented dataset (32000 sequences for training and 8000 sequences for validation), and for the augmented original dataset with splitting (32000 sequences for training and 8000 sequences for validation). CD-HIT (Cluster Database at High Identity with Tolerance) from Biopython library is used for clustering the sequences in both training and validation datasets by efficiently grouping similar sequences together. High similarity pairs of *k-mer* motifs (k = 6) were detected using Jaccard similarity greater than 0.80, 0.90, 0.95, and 0.99. The semantic leakage of training sequences to the validation sequences was calculated by using the cosine similarity (higher than 0.95) of embeddings obtained from tokens of each sequence. The pairs of sequences with cross-split k-nearest neighbors (k = 5) were found using the NearestNeighbors library from sklearn package.

Dataset	Exact duplicates	CD-HIT cluster leakage	*k-mer* leakage (k = 6)	Semantic leakage	Cross-nearest-neighbor leakage
Original	0	0	100	538	1899
Augmented Cutoff 80%	0	0	361	7982	26789
Cutoff 90%	0	0	86	7995	26791
Cutoff 95%	0	0	14	7982	26791
Cutoff 99%	0	0	0	4623	26791
Augmented original with splitting	0	0	0	17	88

Detailed characterization of the peptide representation space

The quantitative analysis reported in Table S2 provides a detailed characterization of the geometric and statistical structure of the peptide representation space, as derived from the multimodal embedding framework integrating ESM-based global semantics and local sequence features. The intra-class diversity, inter-class similarity, and Silhouette score collectively describe how allergen (Class 1) and non-allergen (Class 0) peptides are distributed within this high-dimensional latent space, while permutation testing and bootstrap confidence intervals provide rigorous statistical validation of the observed differences.

In the original dataset, the intra-class diversity is moderately higher for allergen peptides (0.254) compared to non-allergens (0.230), indicating that allergenic peptides occupy a slightly broader region of the embedding manifold. This suggests that allergenicity arises from a more heterogeneous set of sequence–structure patterns, consistent with the notion that multiple distinct epitope configurations can trigger immune responses. The inter-class similarity of 0.649 is relatively high, indicating substantial overlap within allergen and non-allergen representations. Geometrically, this reflects a shared backbone of physicochemical and evolutionary constraints captured by the ESM embeddings. The Silhouette score is a metric used to evaluate the quality of clustering techniques, ranging from -1 to 1. It measures how similar a sequence is to its own cluster (cohesion) versus other clusters (separation). A high Silhouette score near 1 indicates well-separated and dense clusters, while negative values indicate incorrect clustering. The Silhouette score of 0.158, although modest, is positive, indicating that clusters are weakly but meaningfully separable. The permutation test is a non-parametric, exact statistical method that evaluates the significance of a hypothesis by shuffling group labels to simulate a large number of rearrangements (n = 1000) of the observed data. It tests if the observed difference between groups is rare, calculating a p-value by comparing the original statistic to this reshuffled null distribution. Importantly, the permutation test yields a statistically significant result ($p = 0.000$), demonstrating that the observed difference in intra-class diversity is unlikely to arise by chance. The bootstrap confidence intervals (CI) for intra-class diversity are used to calculate the robustness of diversity difference. Non-overlapping

bootstrap CIs means a statistically robust diversity difference. The bootstrap confidence intervals for Class 0 ([0.2160, 0.2439]) and Class 1 ([0.2508, 0.2560]) are non-overlapping, confirming the robustness of this diversity difference. From a biological perspective, this implies that allergenic peptides are intrinsically more diverse in sequence and structural features than non-allergenic peptides, even in the absence of augmentation.

In the augmented dataset, a pronounced shift in the geometric structure is observed. The intra-class diversity for Class 0 increases substantially to 0.453, while Class 1 remains relatively stable at 0.255. This indicates that augmentation disproportionately expands the diversity of non-allergen peptides, likely through the introduction of recombined or extended motifs that increase sequence variability. Concurrently, the inter-class similarity decreases to 0.300, suggesting that the two classes become more separated in the embedding space. This reduction in inter-class similarity is consistent with a more distinct allergen versus non-allergen manifold, supporting the notion of a clearer decision boundary. However, the Silhouette score increases to 0.315, indicating improved clustering structure despite the introduction of synthetic variability. Notably, the permutation test statistic becomes negative (−0.1983, $p = 0.000$), reflecting a reversal in the relative diversity ordering between classes, where non-allergens now exhibit greater dispersion than allergens. The bootstrap confidence intervals remain non-overlapping (Class 0: [0.4515, 0.4553]; Class 1: [0.2523, 0.2581]), confirming that this inversion is statistically robust. This suggests that augmentation alters the intrinsic geometry of the dataset, potentially enhancing separability but also introducing artificial expansion of the non-allergen space.

The most refined representation is observed in the augmented dataset with test dataset splitting before augmentation. Here, intra-class diversity further increases for Class 0 (0.535) and moderately for Class 1 (0.277), indicating a continued expansion of the embedding space, particularly for non-allergens. The inter-class similarity decreases further to 0.214, which is indicative of a more distinct allergen space, consistent with improved class separability in the latent manifold. This aligns with the interpretation that low inter-class similarity corresponds to well-separated functional regions associated with allergenicity. The Silhouette score (0.312)

remains high and comparable to the augmented dataset, suggesting that clustering quality is preserved even after enforcing strict data independence through test dataset splitting before augmentation. The permutation test again yields a statistically significant result (−0.258, p = 0.000), confirming that the difference in diversity between classes is highly unlikely under the null hypothesis. Crucially, the bootstrap confidence intervals remain non-overlapping (Class 0: [0.5329, 0.5364]; Class 1: [0.2733, 0.2801]), providing strong evidence that the observed diversity differences are stable and not driven by sampling variability.

From a broader perspective, these results illustrate how augmentation and dataset splitting strategies reshape the geometry of peptide representation space. The original dataset reflects a biologically realistic but partially overlapping manifold, where allergenicity is embedded within shared sequence constraints. Augmentation expands this manifold, particularly for non-allergens, increasing diversity and reducing inter-class similarity, thereby facilitating classification. However, the augmented dataset with test dataset splitting before augmentation achieves both high separability and statistical robustness without introducing leakage, as evidenced by the combination of low inter-class similarity, stable Silhouette scores, significant permutation tests, and non-overlapping confidence intervals. Collectively, these findings support the conclusion that the multimodal model operates within a well-structured embedding space where allergenicity emerges as a distinct yet complex region characterized by heterogeneous sequence features and separable latent representations.

Table S2. The intra-class diversity for classes 0 and 1, the inter-class similarity, and the Silhouette score. Low inter-class diversity means distinct allergen space. The permutation test (with p-value) evaluates the significance of a hypothesis by shuffling group labels to simulate a large number of rearrangements (n = 1000) of the observed data. The bootstrap confidence intervals (CI) for intra-class diversity are used to calculate the robustness of diversity difference. Non-overlapping bootstrap CIs means a statistically robust diversity difference.

Dataset	Intra-class diversity (Class 0)	Intra-class diversity (Class 1)	Inter-class similarity	Silhouette score	Permutation test (p-value)	Diversity 95% CI (Class 0)	Diversity 95% CI (Class 1)
Original	0.230	0.254	0.649	0.158	0.0230 (0.000)	0.2160 0.2439	0.2508 0.2560
Augmented	0.453	0.255	0.300	0.315	-0.1983 (0.000)	0.4515 0.4553	0.2523 0.2581
Augmented original with splitting	0.535	0.277	0.214	0.312	-0.258 (0.000)	0.5329 0.5364	0.2733 0.2801

Performance metrics for training and validation

The performance metrics reported in Table S3, the training (blue line) and validation (orange line) losses in Figure S1, and the confusion matrices of training (left panel, A) and validation (right panel, B) in Figures S2-S4 provide a comprehensive evaluation of the optimized multimodal deep learning framework integrating Evolutionary Scale Modeling (ESM2-t6-8M-UR50D) embeddings with a hybrid Temporal Convolutional Network (TCN) and Layer-Normalized LSTM architecture for allergen peptide classification. The simultaneous consideration of accuracy, precision, recall, F1-score, Matthews Correlation Coefficient (MCC), and Cohen's Kappa enables a robust assessment of predictive performance, calibration, and reliability under both balanced and potentially imbalanced conditions. Importantly, the distinction between training (upper values) and validation (lower values) allows for a detailed analysis of generalization behavior across the three dataset configurations: original, augmented, and augmented dataset with test dataset splitting before augmentation.

For the original dataset, the model achieves very high training performance (accuracy = 0.979, precision = 0.962, recall = 0.986, F1-score = 0.968, MCC = 0.990, Cohen's Kappa = 0.990), indicating that the architecture effectively captures both global semantic information from ESM embeddings and local sequence dependencies through TCN–LSTM processing. However, a noticeable decline is observed in validation performance (accuracy = 0.988, precision = 0.979, recall = 0.899, F1-score = 0.812, MCC = 0.899, Cohen's Kappa = 0.808). This discrepancy suggests the presence of moderate generalization limitations, particularly reflected in the reduced recall and F1-score. From a statistical perspective, the drop in MCC and Kappa indicates that the classifier, while still highly accurate, exhibits some imbalance in predictive consistency when exposed to unseen data. Biologically, this may reflect the limited diversity and size of the original dataset, which constrains the model's ability to fully capture the heterogeneous nature of allergenic motifs and sequence contexts.

In contrast, the augmented dataset demonstrates near-perfect performance across both training and validation sets, with all metrics approaching unity (accuracy, precision, recall, F1-score, MCC, and Kappa ≈ 0.999). This dramatic improvement reflects the impact of data augmentation in expanding the effective sequence space, enabling the model to learn a richer and more generalized representation of allergenicity-related features. The convergence of training and validation metrics suggests excellent apparent generalization; however, when interpreted alongside the previously observed increases in motif-level and semantic similarity, this performance likely reflects a denser and more redundant representation space. From a geometric standpoint, augmentation increases the sampling density of the embedding manifold, effectively smoothing decision boundaries and facilitating classification. Nevertheless, such near-perfect agreement across all metrics raises concerns regarding potential information redundancy or subtle leakage at the motif or embedding level, which can artificially inflate performance despite the absence of exact sequence duplication.

The most compelling results are observed for the augmented dataset with test dataset splitting before augmentation, where performance remains essentially perfect across all metrics for both training and validation (accuracy, precision, recall, F1-score, MCC, and Kappa ≈ 0.999–1.000). Unlike the fully augmented dataset, this configuration incorporates strict controls to eliminate motif-level and semantic leakage, as demonstrated in the leakage analysis. The preservation of near-perfect performance under these constraints provides strong evidence that the model's predictive capability is not driven by trivial redundancy but rather by its ability to learn biologically meaningful representations of allergenicity. The consistently high MCC and Cohen's Kappa values are particularly important, as they confirm robust classification performance even under strict statistical criteria that account for chance agreement and class imbalance. From a machine learning perspective, this indicates that the model has successfully learned a stable decision boundary in the multimodal feature space that generalizes to independent data. From a biological standpoint, it suggests that allergenicity is encoded in reproducible sequence–structure patterns that can be captured by the combined ESM–TCN–LSTM architecture.

Table S3. Performance statistical metrics (accuracy, precision, recall, F1 score, Matthews correlation coefficient (MCC), and Cohen´s kappa) of training (upper) and validation (lower) of the original, augmented, and augmented original datasets after optimization of the deep learning model (TCN + LSTM) to classify allergen peptides using an Evolutionary Scale Modeling (ESM) language model (esm2_t6_8M_UR50D) to capture both global semantic and local structural features.

Dataset	Accuracy	Precision	Recall	F1 score	MCC	Cohen's Kappa
Original	0.979<br>0.962	0.986<br>0.968	0.990<br>0.990	0.988<br>0.979	0.899<br>0.812	0.899<br>0.808
Augmented	0.999<br>0.999	0.999<br>0.999	0.999<br>0.999	0.999<br>0.999	0.999<br>0.998	0.999<br>0.998
Augmented original with splitting	0.999<br>0.999	1.000<br>1.000	0.999<br>0.999	0.999<br>0.999	0.999<br>0.999	0.999<br>0.999

Additionally, the multimodal architecture integrates both global semantic representations (ESM embeddings) and local sequence features (TCN), which extends beyond simple motif counting approaches such as *k-mer* frequency models. Nevertheless, we agree that simpler baselines are valuable; therefore, the model's performance on the original dataset provides a conservative benchmark independent of augmentation. Also, it is important to mention that much simpler techniques such as counting *k-mers* or finding sequence motifs with one-hot encoding by using Random Forests, Extra Trees, Support Vectors, Gradient Boosting, KNeighbors, and XGBoost models do offer good performance in accuracy and precision (see Table S4 for counting *k-mers* and Table S5 for finding sequence motifs with one-hot encoding), but they do not offer good *k-mer* motif interpretability due to the use of local sequence features and the need of global semantic representation that are not used in these simple methods. Interestingly, Tables S4 and S5 show excellent metrics for all models in both training and validation, except ExtraTrees, Support Vectors, and KNighbors that do not achieve good MCC and Cohen´s kappa. However, it is very important to emphasize these simple methods do use local sequence features and do not use a global semantic representation, i.e., they do not differ peptide sequences PPPQQQ from PQPQPQ, for example, which in our case is crucial for immunological interpretation. In fact, although the simple models perform well in accuracy and precision metrics, they fail in explainability and interpretability using Anchor, LIME, and SHAP methods.

Table S4. Performance statistical metrics (accuracy, precision, recall, F1 score, Matthews correlation coefficient (MCC), and Cohen´s kappa) of training (upper) and validation (lower) of the original dataset after optimization of simple models (Random Forests, Extra Trees, Support Vectors, Gradient Boosting, KNeighbors, and XGBoost) to classify allergen peptides using counting *k-mers* to capture only local structural features.

Model	Accuracy	Precision	Recall	F1 score	MCC	Cohen's Kappa
Random Forest	1.000<br>1.000	1.000<br>1.000	1.000<br>1.000	1.000<br>1.000	1.000<br>1.000	1.000<br>1.000
Extra Trees	1.000<br>0.964	1.000<br>0.961	1.000<br>1.000	1.000<br>0.980	1.000<br>0.821	1.000<br>0.805
Support Vectors	0.956<br>0.941	0.965<br>0.947	0.986<br>0.988	0.975<br>0.967	0.779<br>0.691	0.775<br>0.676
Gradient Boosting	1.000<br>0.998	1.000<br>1.000	1.000<br>0.998	1.000<br>0.999	1.000<br>0.992	1.000<br>0.992
KNeighbors	0.965<br>0.943	0.985<br>0.966	0.975<br>0.970	0.980<br>0.968	0.840<br>0.725	0.839<br>0.725
XGBoost	1.000<br>0.995	1.000<br>0.998	1.000<br>0.996	1.000<br>0.997	1.000<br>0.975	1.000<br>0.975

Table S5. Performance statistical metrics (accuracy, precision, recall, F1 score, Matthews correlation coefficient (MCC), and Cohen´s kappa) of training (upper) and validation (lower) of the original dataset after optimization of simple models (Random Forests, Extra Trees, Support Vectors, Gradient Boosting, KNeighbors, and XGBoost) to classify allergen peptides finding sequence motifs with one-hot encoding to capture only local structural features.

Model	Accuracy	Precision	Recall	F1 score	MCC	Cohen's Kappa
Random Forest	1.000<br>0.996	1.000<br>0.996	1.000<br>1.000	1.000<br>0.998	1.000<br>0.983	1.000<br>0.983
Extra Trees	1.000<br>0.962	1.000<br>0.959	1.000<br>1.000	1.000<br>0.979	1.000<br>0.811	1.000<br>0.794
Support Vectors	0.973<br>0.946	0.972<br>0.944	0.998<br>0.998	0.985<br>0.970	0.867<br>0.721	0.860<br>0.690
Gradient Boosting	1.000<br>0.998	1.000<br>1.000	1.000<br>0.998	1.000<br>0.999	1.000<br>0.992	1.000<br>0.992
KNeighbors	0.962<br>0.937	0.973<br>0.949	0.985<br>0.982	0.979<br>0.965	0.815<br>0.676	0.814<br>0.667
XGBoost	1.000<br>0.996	1.000<br>0.998	1.000<br>0.998	1.000<br>0.998	1.000<br>0.983	1.000<br>0.983

Evaluation of Machine Learning and Deep Learning Models, and Explanation models

Traditional machine learning approaches, such as Random Forests, Extra Trees, Support Vectors, Gradient Boosting, KNeighbors, and XGBoost, exhibit intrinsic limitations when applied to peptide sequence analysis because these models fundamentally operate as pattern-matching systems over fixed feature spaces rather than as architectures capable of learning the contextual and compositional grammar underlying biological sequences. Peptides are not merely collections of independent amino acids; instead, their biochemical activity emerges from highly context-dependent interactions in which the functional contribution of a residue is determined by its sequential and structural environment. Consequently, the predictive failure of conventional machine learning methods cannot be attributed solely to insufficient data availability, but rather to an inherent representational inadequacy. These models rely predominantly on handcrafted encodings, particularly one-hot representations, which transform sequences into sparse position-specific vectors that destroy semantic continuity and prevent the learning of higher-order biological relationships.

One major limitation of simple methods arises from the inability of traditional models to efficiently capture positional interdependence and non-linear combinatorial logic within peptide sequences. Biological activity frequently depends on the simultaneous occurrence of specific residues at distinct positions, analogous to logical conjunctions or XOR-like relationships. An XOR-like relationship (Exclusive OR) refers to a scenario where an outcome or relationship is only true or present if exactly one of two conditions is met, but not both, and not neither. It operates on the principle of one or the other, but not both. For example, a peptide may only exhibit activity if lysine occupies position 1 while glutamate occupies position 5, whereas the presence of either residue alone is insufficient. Decision-tree-based algorithms and linear hyperplane methods are poorly suited for representing such conjunctions because they evaluate features largely independently. In practice, these methods require explicit construction of interaction terms to encode combinations such as (Position 1 = K AND Position 5 = E). However, for peptide sequences containing dozens of positions and twenty possible amino acids per residue, the combinatorial search space becomes exponentially large,

rendering exhaustive feature engineering computationally infeasible. As a consequence, these algorithms often identify marginal positional effects while failing to recognize the biologically meaningful joint dependency responsible for activity.

A second critical issue of simple methods is the curse of dimensionality induced by one-hot encoding schemes. In a typical representation of a 40-residue peptide, each amino acid position is encoded independently, generating a highly sparse vector space with hundreds of binary dimensions. Within this representation, peptides that differ by a single conservative mutation may appear nearly as dissimilar as peptides containing radical physicochemical substitutions. For instance, replacing leucine with isoleucine, both hydrophobic residues with similar steric properties, is treated equivalently to replacing leucine with arginine, a positively charged residue with markedly distinct biochemical behavior. Distance-based relationships in this feature space therefore fail to preserve chemical semantics. Traditional machine learning algorithms operating on such representations cannot learn smooth functional gradients associated with biochemical similarity; instead, they are restricted to detecting exact coordinate matches. This limitation prevents the construction of biologically meaningful latent manifolds in which related peptide sequences occupy neighboring regions of representational space.

Another important limitation of simple methods concerns the absence of positional transfer learning. In one-hot encoded representations, the feature corresponding to alanine at position 15 is entirely independent from alanine at position 16, despite the identical biochemical identity of the residue. Consequently, conventional machine learning models cannot generalize motifs across different sequence locations. A functional motif identified at one region of the sequence is not automatically recognized when shifted to another location because the corresponding features occupy unrelated dimensions in the input space. This deficiency is particularly problematic for peptide analysis because many biologically relevant motifs exhibit positional flexibility and variable lengths. Capturing such patterns using simple models would require extensive manual engineering of motif descriptors, which introduces strong prior assumptions and reduces the model’s ability to discover previously unknown sequence patterns.

Traditional machine learning methods are also fundamentally limited in their ability to model long-range interactions between distant residues. Peptide stability, aggregation propensity, receptor binding, and secondary structure formation frequently depend on interactions between amino acids that are far apart in primary sequence but spatially proximal in three-dimensional conformations. Examples include salt bridges, hydrogen-bonding networks, and hydrophobic packing interactions that stabilize helices or β-sheet assemblies. Decision trees can only evaluate these dependencies indirectly through hierarchical branching structures, requiring multiple sequential splits to approximate a single interaction. In high-dimensional sparse spaces with limited biological datasets, these deeper interaction pathways are rarely explored effectively because early splits are dominated by spurious or locally informative features. Ensemble averaging further dilutes weak but biologically important dependencies, causing long-range interactions to be obscured by noise. As a result, traditional models are biased toward superficial positional correlations rather than mechanistic sequence relationships.

Collectively, these limitations demonstrate that classical machine learning algorithms primarily memorize local patterns and shallow positional biases rather than infer the semantic grammar governing peptide function. In contrast, modern deep learning architectures such as convolutional neural networks, recurrent neural networks, transformers, attention-based models, and hybrid neural networks as shown in this study, are specifically designed to learn contextual and compositional representations directly from sequential data. These architectures encode amino acid relationships in dense latent spaces where biochemical similarity, positional flexibility, and long-range dependencies can emerge naturally through training. Convolutional filters enable the detection of transferable motifs independent of exact sequence location, while attention mechanisms dynamically model interactions between distant residues. Consequently, deep learning approaches provide a fundamentally more expressive framework for representing peptide semantics and functional landscapes.

This conceptual distinction can be experimentally demonstrated using synthetic datasets specifically designed to contain combinatorial sequence rules. For example, a classification task in which activity depends exclusively on the conjunction of residues at two distant positions, such as (Pos10 = K and Pos30 = E) or (Pos10 = R and Pos30 = D), typically causes Random Forest and Support Vector Machine models trained on one-hot encodings to perform near random expectation, especially in the presence of noise sequences. In contrast, even relatively simple neural architectures incorporating convolutional or attention-based mechanisms can solve the problem efficiently because they naturally learn contextual feature interactions without requiring explicit manual specification. Such experiments provide compelling evidence that the superiority of deep learning models in peptide prediction tasks originates not merely from increased parameterization, but from their ability to capture the hierarchical and contextual grammar intrinsic to biological sequences.

Table S6 presents the performance of the optimized multimodal ESM–TCN–LSTM model on an independent testing dataset that was separated prior to any augmentation or dataset splitting procedures. This experimental design is particularly critical, as it ensures that the test set remains fully isolated from the training and validation pipelines, thereby providing an unbiased evaluation of the model's true generalization capability. This table reports sequence-level predictions alongside ground truth annotations ("Allergen" vs "Predicted"), allowing for a direct inspection of agreement between experimental labels and model outputs at the individual peptide level.

A striking observation from the table is the high degree of concordance between the "Allergen" and "Predicted" columns, particularly for sequences annotated as allergenic ("Yes"). The vast majority of allergenic peptides are correctly classified, indicating that the model exhibits strong sensitivity (recall) toward allergenic sequences. This suggests that the multimodal architecture effectively captures the underlying sequence–structure determinants of allergenicity, including conserved motifs, charge distributions, cysteine patterns, and higher-order contextual dependencies encoded in the ESM embeddings. From a mechanistic standpoint, this

performance reflects the model's ability to integrate global evolutionary constraints (captured by the transformer-based ESM representations) with local sequential patterns (modeled through TCN and LSTM layers), enabling robust identification of diverse allergenic signatures across structurally heterogeneous peptides.

For non-allergenic peptides ("No"), the agreement is also high, with most sequences correctly predicted as non-allergens. However, a small number of discrepancies are observed, where non-allergenic sequences are predicted as allergenic (false positives). These cases are particularly informative from a biological and representational perspective. Inspection of these sequences reveals that they often contain repetitive motifs, charged residues, or structural patterns that may resemble known allergenic features in the embedding space. Consequently, the model assigns them to regions of the latent manifold associated with allergenicity. While this results in a slight reduction in specificity, it also indicates that the model is sensitive to borderline or ambiguous sequence features that may share partial similarity with allergenic epitopes. Importantly, no systematic bias toward false negatives is evident, suggesting that the model prioritizes minimizing missed allergenic predictions, which is a desirable property in safety-critical applications.

The high agreement between true and predicted labels implies that the decision boundary learned by the model is well-aligned with the intrinsic structure of the peptide embedding space. Allergenic sequences cluster within a distinct region characterized by conserved biochemical and evolutionary features, while non-allergenic sequences occupy a separate but partially overlapping region. The few misclassified instances likely reside near the boundary between these regions, where local similarity to allergenic motifs influences the model's prediction. This interpretation is consistent with the observed embedding space visualizations (UMAP and t-SNE) (see Figures S5 and S6), where class separation is strong but not absolute due to the continuous nature of protein sequence space. The results in Table S6 provide strong evidence that the model generalizes effectively to an independent test set, with a high level of agreement between experimental annotations and predicted labels. The minimal number of discrepancies, combined with their plausible biological interpretation, supports the conclusion that the model captures meaningful and transferable features of

allergenicity rather than relying on dataset-specific artifacts or redundancy. This reinforces the robustness and translational potential of the proposed multimodal framework for peptide allergen prediction.

Table S6. Comparison of Lime explanations of the RF (first row) and TCN/LSTM (second row)) models using the original dataset.

Real	Predicted	kmer_1	kmer_2	kmer_3	kmer_4	kmer_5	kmer_6	kmer_7	kmer_8	kmer_9	kmer_10
1	Allergen	ITCGQV	ACNCLK	PYKISP	AITCGQ	AACNCL	DRQAAC	GKCGVN	QAACNC	ACCNGV	LPYKIS
	Allergen	AAITCGQVVSK**LTNCLSYLRSGG**TVSTACCNGVTSLNKM**ANSTSD**RQAACNCLKSAYKSISGIKLQYSQSLAGKCGVNLP**YKISPD**IDCSKVK									
1	Allergen	VCPTLK	HLRACQ	LRACQQ	QCCNEL	RACQQW	KTMPGP	QHLRAC	QQAQHL	IYQTAT	QQGQQG
	Non-Allergen	AGPFRFPRCRKEFQQAQHLRACQQWLHKQAMQSGSGPQPQGPQQRPPLLQQCCNELHQEEPLCVCPTLKGASKAVKQQIRQQGQQQGQQGQQLQHEISRIYQTATHLPRVCNIPRVSICPFQKTMPGPS									
1	Non-Allergen	FRTTIT	RTTITT	AFRTTI	AAFRTT	AAAFRT	EIDEDI	MEEMVQ	CEGLRQ	QCEGLR	IDNPRR
	Non-Allergen	AALLVALLFVANAAAFRTTITTMEIDEDIDNPRRRGEGCREQIQRQQNLNHCQYYLRQQSRSGGYDEDNQRQHFRQCC**QQLSQM**DEQCQCEGLRQVVRRQQQQQGLRGEEMEEMVQSARDLPNECGISSQRCEIRRSWF									
1	Allergen	RRERGR	RQGDSS	RCCDEL	RERGRQ	RGRQGD	QGDSSS	MRRERG	GRQGDS	DLDVSG	ERGRQG
	Allergen	AHAS**AMRRER**GRQGDSSSCERQVDGVNLKPCE**QHIMQR**IMGEQEQYDSY**NFGSTR**SSDQQQRCCDELNEMENTQRCMCEALQQIMENQCDGLQDRQMVQH**FKRELM**NLPQQCNFGAPQRCDLDVSGGRC									
1	Allergen	GPFNFR	PFNFRF	TVEKGS	MAEVEL	FDDVVP	VFDDVV	ADFKVG	FVSEKG	GSNEWL	NVFDDV
	Allergen	AAPVEFTVEKGSDEKNLAL**SIKYNK**EGDSMAEVELK**EHGSNEWLALKK**N**GDGVWE**IKSDKPLKGPFNFRFVSEKGMRNVFDDVVPADFKVGTTYKPE									
1	Allergen	RRERGR	RQGDSS	RCCDEL	RGRQGD	RERGRQ	GRQGDS	QGDSSS	MRRERG	DLDVSG	ERGRQG
	Allergen	AKSTILVALLALVLVAH**ASAMRR**ERGRQGD**SSSCER**QVDRVNL**KPCEQH**IMQRIMGEQEQY**DSYDIR**STRSSDQQQRCCDELDQMENTERCMCEALQQIMENQCDRLQDRQM**VQQFKR**ELMNLPQQCNFRAPQRCDLDVSGGRC									
1	Allergen	GRKPEG	FNIKAT	GTLDFT	LDFTCS	KPEGTY	CSAQAD	YSCGEN	TCSAQA	RKPEGT	FTCSAQ
	Allergen	ALFAAAGLAA**AAPFEA**RQADASCPVST**QGDYVW**KISEF**SGRKPE**GTYYNSLSFNIKAT**NKGTLD**FTCSAQADKLEDDKFYSCGENSFMSF**AFQSDR**NGLLFRQDVSDEITYVATAT**LPNYCH**AGGNGPKDFVCQGVS									
1	Allergen	LKLFLQ	ELKLFL	FLKAGD	LFLQNF	KLFLQN	TDAETK	EELKLF	ETKAFL	KAFLKA	LTDAET
	Allergen	ACAHLCKEAD**LKTALE**ACKAAD**SFNFKT**FFHTLGFASKXXDDVKKXXXXV**LDQDASGFLEVE**ELKLFLQNFCPK**XXXLTD**AETKAFLKAGD**ADGDGM**LGLDEFAVLVKQ									
1	Non-Allergen	VEFPGS	PGMLGE	AAIETI	TLDNVD	EVDLWT	YFDVSA	YAYPTD	HEVDLW	EVQFGG	SWVGNW
	Non-Allergen	ASVTFWT**LDNVDR**TLVFTGNPGSAAIETITVGPAENTTVEFPG**SWVGNW**YAYPTDAEDVPGMLGE**VQFGGW**NGLTYFDVSAIVNPTDHD**NVKQMW**PAESR**KPMSGC**EV**FPCDNA**YWLPDDIQTKVT[HEVDLW]TTLGAGSTGLTF									
1	Allergen	VYCDTC	YCDTCR	RSANAL	KVSLTS	SANALG	NGEASD	SNNGEA	VSLTSN	GTQTFK	NALGFM

Allergen	DDVAKPPVSQFHIQ**GLVYCD**TCRIQFMTR**VSTIME**G**ATVKLE**CRNITAGTQTFKAEAVTDKVGQYSIPVNGDFEDDICEIELVKSPNSD**CSEIPH**DVYAKQSAKVSLTSNNGEASDIRSANALGFMRKEPLKECPEVSKEPLDMYDVKAN

Table S7. Comparison of Shap explanations of the RF (first row) and TCN/LSTM (second row)) models using the original dataset.

Real	predicted	kmer_1	Shap 1	kmer_2	Shap 2	kmer_3	Shap 3	kmer_4	Shap 4	kmer_5	Shap 5
1	Allergen	ITCGQV	9273	ACNCLK	6326	AACNCL	0	AAITCG	0	ACCNGV	0
	Allergen	AAITCGQVVSKL**TNCLSY**LRSGGTVSTACCNGVTSLNKMANSTSDRQAACNCLKSAYKSISGIKLQYSQSLAGKCGVNLPYKISPDIDCSKVK									
1	Allergen	VCPTLK	2535	HLRACQ	2193	RACQQW	1713	ACQQWL	0	AMQSGS	0
	Non-Allergen										
1	Non-Allergen	LLFVAN	-5135	AAAFRT	0	AAFRTT	0	AALLVA	0	AFRTTI	0
	Non-Allergen										
1	Allergen	AHASAM	0	ALQQIM	0	AMRRER	0	APQRCD	0	ASAMRR	0
	Allergen	**AHASAM**RRERGR**QGDSSS**CERQVDGVNLKPCEQHIMQRIMGEQEQYDSYNFGSTRSSDQQQRCCDELNEMENTQRCMCEALQQIMENQCDGLQDRQMVQHFKRELMNLPQQCNFGAPQRCDLDVSGGRC									
1	Allergen	VPADFK	-1328	ADFKVG	0	AEVELK	0	ALKKNG	0	ALSIKY	0
	Allergen	**AAPVEF**TVEK**GSDEKN**LALSIKYNKEGDSM**AEVELK**EHGSNEWLALKKNGDGVWEIKSDKPLKGPFNFRFVSEKGMRNVFDDVVPADFKVGTTYKPE									
1	Allergen	RRERGR	3863	RCCDEL	3511	HIMQRI	-868	MNLPQQ	-615	AHASAM	0
	Allergen	AKSTILVALLALVL**VAHASA**MRRERGR**QGDSSS**CERQVDRVNLKPCEQHIMQRIMGEQEQYDSYDIRSTRSSDQQQRCCDELDQMENTERCMCEALQQIMENQCDRLQDRQMVQQFKRELMNLPQQCNFRAPQRCDLDVSGGRC									
1	Allergen	GRKPEG	3245	GTLDFT	2963	PEGTYY	1992	CSAQAD	1576	YSCGEN	1053
	Allergen	**ALFAAAGLAAAA**PFEARQADASCPVSTQGDYVWKISEFSGRKPEGTYYNSLSFNIKATNKGTLDFTCSAQADKLEDDKFYSCGENSFMSFAFQSDRNGLLFRQDVSDEITYVATATLPNYCHAGGNGPKDFVCQGVS									
1	Allergen	LKLFLQ	4648	LFLQNF	4451	ELKLFL	297	AGDADG	-1933	AADSFN	0
	Allergen	ACAH**LCKEAD**LKTALEACK**AADSFN**FKT**FFHTLG**FASKXXDDVKKXXXVLDQDASGFLEVEELKLFLQNFCPKXXXLTDAETKAFLKAGDADGDGMLGLDEFAVLVKQ									
1	Non-Allergen										

Real	Predicted										
	Non-Allergen	ASVTFWTLDNVDRTLVFTGNP**GSAAIE**TITVGPAENTTVEFPGS**WVGNWY**AYPTDAEDVPGMLGEVQFGGWNGLTYFDVSAIVNPTDHDNVKQMWPAESRKPMSGCEVFPCDNAYWLPDDIQTKVTHEVDLWTTLGAGSTGLTF									
1	Allergen	LVYCDT	-467	AEAVTD	0	AGTQTF	0	AKQSAK	0	AKVSLT	0
	Allergen	**DDVAKP**PVSQFHIQGLVYCDTCRIQFMTR**VSTIME**GATVK**LECRNI**TAGTQTFKAEAVTDKVGQYSIPVNGDFEDDICEIELVKSPNSDCSEIPHDVYAKQSAKVSLTSNNGEASDIRSANALGFMRKEPLKECPEVSKEPLDMYDVKAN									

Table S8. Comparison of Lime explanations of the RF (first row) and TCN/LSTM (second row)) models using the augmented dataset.

Real	Predicted	kmer_1	kmer_2	kmer_3	kmer_4	kmer_5	kmer_6	kmer_7	kmer_8	kmer_9	kmer_10
1	Non-Allergen	AAITCG	AITCGQ	ITCGQV	TCGQVV	CGQVVS	GQVVSK	QVVSKL	VVSKLT	VSKLTN	SKLTNC
	Allergen	AAITC**GQVVSK**LTNCLSYLRSGGTVSTACCNG**VTSLNK**M**ANSTSD**RQAACNCLKSAYK**SISGIK**LQYS**QSLAGK**CGVNLPYK**ISPDID**CSKVK									
1	Non-Allergen	AGPFRF	GPFRFP	PFRFPR	FRFPRC	RFPRCR	FPRCRK	PRCRKE	RCRKEF	CRKEFQ	RKEFQQ
	Non-Allergen	A**GPFRFP**RCRKEFQQAQHL**RACQQW**LHKQAMQSGSGPQPQ**GPQQRP**PLLQQCCNE**LHQEEP**LCVCPTLKGASKAV**KQQIRQ**QGQQQGQQGQQLQHEISRIYQTATHLPRVCNIPR**VSICPF**QKTMPGPS									
1	Non-Allergen	AALLVA	ALLVAL	LLVALL	LVALLF	VALLFV	ALLFVA	LLFVAN	LFVANA	FVANAA	VANAAA
	Non-Allergen	AALLVALLFVANAAAFRTTITTMEIDEDIDNPRRRGEGCREQIQRQQNLNHCQYYL**RQQSRS**GGYDED**NQRQHF**RQCCQQLSQMDEQCQCE**GLRQVV**RRQQQQQGLRGE**EMEEMV**QSARDLPNECGISSQRCEIRRSWF									
1	Allergen	RRERGR	MRRERG	GRQGDS	RGRQGD	QGDSSS	ERGRQG	RQGDSS	RERGRQ	DRQMVQ	FGAPQR
	Allergen	AHASAMRRER**GRQGDS**SSCE**RQVDGV**NLKPCEQHIMQRIMGEQEQ**YDSYNF**GSTRSSDQQQRCCDELNEMENTQRCMCEALQQIMENQCDGLQDR**QMVQHF**KR**ELMNLP**QQCNFGAPQRCDLDVSGGRC									
1	Non-Allergen	AAPVEF	APVEFT	PVEFTV	VEFTVE	EFTVEK	FTVEKG	TVEKGS	VEKGSD	EKGSDE	KGSDEK
	Allergen	AA**PVEFTV**E**KGSDEK**NLAL**SIKYNK**EGDSMAEVELKEHGSN**EWLALK**KNGDGVWEIKSDKPL**KGPFNFRFVSEK**GMR**NVFDDV**VPADFKVGTTYKPE									
1	Allergen	RRERGR	MRRERG	GRQGDS	QGDSSS	RGRQGD	ERGRQG	RQGDSS	RERGRQ	DQMENT	ERQVDR
	Allergen	AKSTILVALLALVLVAHASAMRRERGRQGDSS**SCERQV**D**RVNLKP**CEQHIMQRIMGE**QEQYDS**YDIRSTRSSDQQQRCCDELDQMENTERCMCEALQQIMENQCDRLQDR**QMVQQF**KRELMNL**PQQCNF**RAPQRCDLDVSGGRC									
1	Non-Allergen	ALFAAA	LFAAAG	FAAAGL	AAAGLA	AAGLAA	AGLAAA	GLAAAA	LAAAAP	AAAAPF	AAAPFE
	Allergen	ALFAAAGLAAAA**PFEARQ**[**ADASCP**VST**QGDYVW**KIS**EFSGRK**PEGTYYN**LSFNIK**ATNKGTLDFTCSAQAD**KLEDDK**FYSCGENSFMSFAFQSDRNGLL**FRQDVS**DEITYVATATLPNYCHA**GGNGPK**DFVCQGVS									
1	Non-Allergen	ACAHLC	CAHLCK	AHLCKE	HLCKEA	LCKEAD	CKEADL	KEADLK	EADLKT	ADLKTA	DLKTAL
	Allergen	ACAHLCKEADLKTALE**ACKAAD**SFNFKTFFH**TLGFAS**KXXDDVKKXXXXV**LDQDAS**GFLEVEE**LKLFLQ**NFCPKXXXLTDAETKAFLKAGDADGDGMLGLDEF**AVLVKQ**									

1	Non-Allergen	ASVTFW	SVTFWT	VTFWTL	TFWTLD	FWTLDN	WTLDNV	TLDNVD	LDNVDR	DNVDRT	NVDRTL
	Non-Allergen	ASVTFWTLDNVDRTLVFTGNPGSAAIETITVGPAENTTVEFPG**SWVGNW**YAYPTDAEDVPGMLGE**VQFGGW**NGLTYFDVSAIVNPTD**HDNVKQ**M**WPAESR**KPM**SGCEVF**P**CDNAYW**LPDDIQTKVT**HEVDLW**TTLGAGSTGLTF									
1	Non-Allergen	VYCDTC	YCDTCR	NNGEAS	TSNNGE	NGDFED	DKVGQY	YSIPVN	QTFKAE	VTDKVG	GTQTFK
	Allergen	DDV**AKPPVS**QFHIQGLVYCDTCRIQFMTRVSTIMEGATVKLECRNITAGTQTFKAEAVTDKVGQYSIPVNGDFEDDICEIELVKS**PNSDCS**EIPHDVYAK**QSAKVS**L**TSNNGE**ASDIRSANA**LGFMRK**EPLKECPEVSKEPL**DMYDVK**AN									

Table S9. Comparison of Shap explanations of the RF (first row) and TCN/LSTM (second row)) models using the augmented dataset.

Real	predicted	kmer_1	Shap 1	kmer_2	Shap 2	kmer_3	Shap 3	kmer_4	Shap 4	kmer_5	Shap 5
1	Non-Allergen										
	Allergen	AAITCG**QVVSKL**TNCLSYLRSGGTVSTACCNGVTSLNKMANSTSDRQAACNCLKSAYKSISGIKLQYSQSLAGKCGVNLPYK**ISPDID**CSKVK									
1	Non-Allergen	AVKQQI	0	PPLLQQ	0	PQGPQQ	0	QGPQQR	0	QQGQQG	0
	Non-Allergen	AGPFRFPRCRK**EFQQAQ**HLRACQQWLHKQAMQSG**SGPQPQ**GPQQRPPLLQQCCNELHQEEPLCVCPTLKGASKAVKQQIRQQGQQQGQQGQQLQHEISRIYQTATHLPRVCNIPRVSICPFQKTMPGPS									
1	Non-Allergen	LRGEEM	0	QQQQQG	0	RQQQQQ	0				
	Non-Allergen	AALLVALLFV**ANAAAF**RTTITTME**IDEDID**NPRRRGEGCREQIQRQQNLNHCQYYLRQQSRSGGYDEDNQRQHFRQCCQQLSQMDEQCQCEGLRQVVRRQQQQQGLRGEEMEEMVQSARDLPNECGISSQRCEIRRSWF									
1	Allergen	RRERGR	5295	GRQGDS	505	MRRERG	3904	RGRQGD	3571	QGDSSS	3258
	Allergen	AHASAMRRERGR**QGDSSS**CERQV[DGVNLK]PCEQHIMQRIMGEQEQYDSYNFGSTR**SSDQQQ**RCCDELNEMENTQRCMCEALQQIMENQCDGLQDRQMVQHFKRELMNLPQQCNFGAPQRCDLDVSGGRC									
1	Non-Allergen										
	Allergen	AAPVEFTVE**KGSDEK**NLALSIKY**NKEGDS**MAEVELKEHGSNEWLALKKNGDGVWEIKSDKPLKGPFNFRFVSEKGMRNVFDDVVPADFKVGTTYKPE									
1	Allergen	RRERGR	5526	MRRERG	4512	GRQGDS	4155	QGDSSS	3463	ERGRQG	3103
	Allergen	AKSTIL**VALLAL**VLVAHASAMRRERGR**QGDSSS**CERQVDRVNLKPCEQHIMQRIMGEQEQYDSYDIRSTR**SSDQQQ**RCCDELDQMENTERCMCEALQQIMENQCDRLQDRQMVQQFKRELMNLPQQCNFRAPQRCDLDVSGGRC									
1	Non-Allergen	KGTLDF	0								

	Allergen	ALFA**AAGLAA**AAPFEAR**QADASC**PVSTQGDYVWKISEFSGRKPEGTYYNSLSFNIKATNKGTLDFTCSAQADKLEDDKFYSCGENSFMSFAFQSDRNGLLFRQDVSDEITYVATATLPNYCHAGGNGPKDFVCQGVS
1	Non-Allergen	
	Allergen	ACAHLCKEADLKTALEAC**KAADSF**NFKTFFHTLGFASKXXDDVKKXXXVLDQDASGFLEVEELKLFLQNFCPKXXXLTDAETKAFLKAGDADGDGMLGLDEFAVLVKQ
1	Non-Allergen	
	Non-Allergen	ASVTFW**TLDNVD**RTLVFTGN**PGSAAI**ETITVGPAENTTVEFPGSWVGNWYAYPTDAEDVPGMLGEVQFGGWNGLTYFDVSAIVNPTDHDNVKQMWPAESRKPMSGCEVFPCDNAYWLPDDIQTKVTHEVDLWTTLGAGSTGLTF
1	Non-Allergen	VYCDTC 0 YCDTCR 0
	Allergen	**DDVAKP**PVSQFHIQGLVYC**DTCRIQ**FMTRVSTIMEGATVKLECRNITAGTQTFKAEAVTDKVGQYSIPVN**GDFEDD**]ICEIELVKS**PNSDCS**EIPHDVYAKQSAKVSLTSNNGEASDIRSANALGFMRKEPLKECPEVSKEPLDMYDVKAN

Comparison of Machine Learning and Deep Learning Models

The comparative analysis presented in Table S10 highlights important methodological and conceptual differences between existing allergen peptide prediction approaches and the framework proposed in this study. The results demonstrate that our model achieves highly competitive, and in several cases superior, predictive performance relative to state-of-the-art methodologies, while additionally providing an explainable artificial intelligence (XAI) framework capable of biologically interpretable decision-making. This combination of predictive accuracy and interpretability represents a significant advancement for allergen peptide analysis, particularly in applications involving dermatological and cosmetic safety assessment, where mechanistic transparency is essential for regulatory and translational relevance.

Traditional allergen prediction models such as AllerTOPv2 and AllergenFP were among the earliest computational frameworks developed for allergenicity prediction and primarily rely on handcrafted physicochemical descriptors and alignment-derived features. Although these approaches demonstrated reasonable predictive performance for their time, their reported accuracy and MCC values remain substantially lower than those obtained by our proposed architecture. Importantly, these earlier methods depend heavily on manually engineered descriptors, which inherently constrain the ability to capture higher-order contextual dependencies and semantic relationships embedded within peptide sequences. Consequently, while such models may identify broad compositional trends, they often fail to represent the non-linear sequence grammar governing allergenic activity, particularly in short peptides where positional interactions and contextual residue dependencies are critical determinants of function.

Similarly, Allerdictor introduced machine learning-based allergen prediction using sequence-derived representations; however, the methodology predates the emergence of modern protein language models and contextual embeddings. As a result, its representational capacity remains limited by shallow sequence encoding strategies that inadequately capture biochemical semantics and long-range dependencies. Moreover, the absence of explainability mechanisms in such frameworks restricts biological

interpretability and reduces confidence in model-driven predictions, particularly in biomedical contexts where understanding the molecular determinants underlying allergenicity is as important as predictive performance itself.

More recent approaches, including AlgPred 2.0 and the pLM4Alg framework, represent substantial methodological improvements by incorporating ensemble learning, deep learning architectures, and pretrained protein language models. Notably, pLM4Alg demonstrates strong performance metrics, including an MCC value comparable to that of our model when evaluated on large datasets. These findings confirm the growing importance of contextual embeddings for protein and peptide representation learning. Nevertheless, despite their strong predictive capabilities, these methods remain largely black-box systems with limited interpretability. In practical biomedical and cosmetic applications, the inability to rationalize predictions in terms of biologically meaningful sequence motifs, residue contributions, or contextual interactions represents a significant limitation. In contrast, our framework integrates explainable AI methodologies directly into the predictive pipeline, enabling the identification of sequence regions and compositional patterns associated with allergenic behavior. This capacity substantially improves the biological interpretability of predictions and facilitates hypothesis generation regarding the molecular mechanisms underlying peptide allergenicity.

An additional strength of the proposed framework lies in its hybrid TCN-LSTM architecture coupled with contextual ESM embeddings. While transformer-derived embeddings already encode rich biochemical and evolutionary information, the integration of temporal convolutional networks and recurrent sequential modeling enables complementary learning of both local compositional motifs and long-range contextual dependencies. This hybrid design is particularly advantageous for short allergenic peptides, whose biological activity frequently emerges from subtle combinations of residues distributed across the sequence. The superior predictive performance observed in both the original and augmented datasets suggests that the proposed model effectively captures contextual sequence semantics beyond simple positional similarity. Importantly, the model maintains strong MCC values, indicating balanced predictive capability even under potentially imbalanced classification scenarios.

The inclusion of explainability further differentiates the proposed framework from nearly all competing methods summarized in Table S10. Among the compared approaches, only AllerStat incorporates explicit interpretability components; however, its predictive performance remains considerably lower, particularly in terms of MCC. This contrast highlights an important trade-off frequently observed in bioinformatics between predictive power and interpretability. Our results demonstrate that this trade-off is not necessarily unavoidable. By combining deep contextual embeddings with explainable learning strategies, the proposed framework achieves both high predictive accuracy and biologically meaningful interpretability, thereby overcoming a longstanding limitation of many deep learning-based peptide prediction systems.

Despite these advantages, several limitations should also be acknowledged. First, direct comparisons between studies remain inherently challenging due to differences in dataset composition, redundancy filtering strategies, peptide length distributions, and evaluation protocols. Some competing models were trained on substantially larger datasets, whereas others focused on full proteins rather than short peptides. Consequently, numerical performance comparisons should be interpreted cautiously. Second, although the augmented dataset produced near-perfect predictive metrics, data augmentation strategies may inadvertently introduce distributional biases or artificial sequence regularities that simplify the classification task. Future work should therefore include evaluation on larger independent external datasets and prospective validation using experimentally characterized allergenic peptides. Finally, while explainability methods provide valuable insights into model behavior, they do not fully resolve the intrinsic complexity of interpreting deep neural representations in biological systems. Accordingly, explainable outputs should be interpreted as supportive mechanistic hypotheses rather than definitive causal evidence.

Table S10. Comparison between our model and Machine Learning-Based Allergen Peptide Prediction Models.

Model	Dataset	Accuracy	Recall	MCC	XAI
Allergen peptide explainer (Our model)	2795(1)+333(0) (Original)	0.979	0.990	0.899	Yes
	40000 (Augmented)	0.999	0.999	0.999	Yes
pLM4Alg (1)	16058	0.951	0.942	0.902	No
AlgPred 2.0 (2)	20150	0.927	0.940	0.860	No
AllerStat (3)	21154	N/A	N/A	0.450	Yes
Allerdictor (4)	42977	N/A	N/A	N/A	No
AllerTOPv2 (5)	4854	0.887	0.867	0.775	No
AllergenFP (6)	4854	0.879	0.868	0.759	No

(1) Du et al., J. Agric. Food Chem. 2024, 72, 1, 752–760. doi: https://doi.org/10.1021/acs.jafc.3c07143

(2) Neelam et al, Brief Bioinform, 2021, 22, bbaa294. doi: https://doi.org/10.1093/bib/bbaa294

(3) Goto et al., J Biol Chem. 2023, 299,104733. doi: https://doi.org/10.1016/j.jbc.2023.104733

(4) Dang and Lawrence, Bioinformatics. 2014, 30,1120-1128. doi: 10.1093/bioinformatics/btu004

(5) Dimitrov et al., J Mol Model. 2014, 20, 2278. doi: 10.1007/s00894-014-2278-5

(6) Dimitrov et al., Bioinformatics, 2014, 30, 846–851. doi: https://doi.org/10.1093/bioinformatics/btt619

Ablation Studies

First, and very important point, the ablation study demonstrates that the proposed hybrid architecture should not be interpreted as a simple additive collection of independent modules designed merely to maximize predictive metrics. Instead, the architecture was intentionally designed to address fundamental representational limitations frequently observed in traditional machine learning approaches applied to peptide analysis. Conventional models operating on one-hot or handcrafted descriptors often fail to capture context-dependent sequence grammar, positional transferability, and long-range residue interactions. By integrating ESM embeddings with TCN-based local pattern extraction, LSTM-based contextual dependency modeling, and feature fusion mechanisms, the proposed framework seeks to construct a richer hierarchical representation of peptide semantics. Interestingly, the ablation results reveal that all model variants achieved relatively strong predictive performance, with only moderate numerical differences across conventional classification metrics such as accuracy, F1-score, and recall. At first glance, these findings may suggest that the individual architectural components contribute similarly to the final classification outcome. However, we emphasize that the primary motivation underlying the proposed hybrid design is not solely incremental metric optimization, but rather the construction of a biologically meaningful representational framework capable of modeling the semantic, contextual, and long-range complexity intrinsic to allergen peptide sequences.

The strong performance observed for the ESM-only configuration confirms the remarkable representational capacity of modern protein language models. Contextual embeddings generated by ESM already encode substantial biochemical, structural, and evolutionary information learned from large-scale protein models. Consequently, even simplified downstream classifiers can achieve high predictive accuracy when operating on these embeddings. Nevertheless, although ESM embeddings provide a powerful latent representation, they do not explicitly enforce task-specific contextual refinement for allergenicity prediction. The downstream sequential modules introduced in our framework were specifically designed to further model compositional sequence dependencies

and contextual interactions relevant to short allergenic peptides, which frequently exhibit subtle semantic patterns not fully captured through global embedding representations alone.

The removal of the TCN module resulted in a modest reduction in MCC and Cohen's Kappa despite maintaining comparable accuracy and F1-score values. This observation suggests that the TCN component contributes primarily to improving robustness and discriminative balance rather than merely increasing overall classification rates. Temporal convolutional layers are particularly effective for capturing local compositional motifs and multi-scale sequence patterns through dilated receptive fields. In the context of allergen peptides, such local motif sensitivity is biologically relevant because allergenic behavior may emerge from short contiguous residue arrangements, conserved physicochemical signatures, or motif-like sequence fragments distributed across peptides of varying lengths. The relatively small performance decrease after removing the TCN module further indicates that ESM embeddings already contain partially encoded local contextual information; however, the TCN still contributes to refining these representations into more task-specific semantic patterns.

Similarly, the removal of the LSTM component did not catastrophically impair predictive performance and, notably, even produced slightly higher MCC and Cohen's Kappa values under the evaluated dataset conditions. While this observation may initially appear counterintuitive, it likely reflects the strong contextual priors already embedded within ESM representations combined with the relatively limited size and sequence length distribution of the dataset. Importantly, however, the inclusion of recurrent contextual modeling should not be interpreted exclusively through the lens of isolated benchmark metrics. The conceptual role of the LSTM module within the hybrid architecture is to provide sequential dependency modeling and contextual memory integration, enabling the network to capture non-local interactions and long-range semantic relationships between residues. Such relationships are biologically important because peptide allergenicity often depends on combinatorial contextual effects rather than isolated amino acid positions. Consequently, even when the numerical performance gains appear modest, the recurrent module contributes to the mechanistic

representational capacity of the architecture and improves its theoretical suitability for modeling sequence grammar and contextual peptide semantics.

The “No Fusion” configuration further highlights the importance of integrative feature learning within the proposed framework. Although performance remained relatively high, the reduction in MCC and Cohen’s Kappa indicates that explicit fusion of complementary representations contributes to more stable and balanced classification. The fusion strategy only enables the integration of local convolutional features, sequential contextual dependencies, and pretrained embedding semantics into a unified latent representation space. This integration is particularly important for allergen peptide analysis because allergenicity is unlikely to arise from a single scale of information alone; rather, it emerges from the interplay between local residue composition, contextual sequence organization, and broader biochemical semantics.

Therefore, although the quantitative differences between ablated configurations remain relatively small, the collective results support the conceptual validity of the hybrid architecture. The importance of the framework lies not only in achieving high predictive accuracy, but also in providing a biologically plausible representational mechanism capable of modeling the semantic and long-range complexity of allergen peptide sequences. This distinction is particularly relevant in explainable AI applications, where the interpretability and mechanistic coherence of learned representations are as important as raw predictive performance.

Table S11. Ablation studies on model components using the original validation dataset.

Model	Accuracy	Precision	Recall	F1-score	MCC	Cohen's Kappa
Full Model	0.957	0.970	0.982	0.976	0.789	0.788
No TCN	0.957	0.962	0.990	0.976	0.783	0.776
No LSTM	0.966	0.976	0.986	0.981	0.834	0.833
No Fusion	0.955	0.960	0.990	0.975	0.773	0.765
ESM only	0.955	0.959	0.992	0.972	0.772	0.762

Deep Learning model performances

Figure S1 presents the training (blue) and validation (orange) loss trajectories over 35 epochs for the multimodal deep learning architecture combining Evolutionary Scale Modeling (ESM2-t6-8M-UR50D) embeddings with a TCN–LSTM framework, providing critical insight into optimization dynamics, convergence behavior, and generalization capacity across the three dataset configurations. These curves reflect the interplay between global semantic representations captured by the pretrained protein language model and local sequence dependencies modeled through temporal convolutional and recurrent layers, thereby serving as a proxy for how effectively the model learns biologically meaningful patterns associated with allergenicity.

In the original dataset (Figure S1A), the training and validation losses exhibit a general downward trend, indicating successful optimization and progressive learning of discriminative features. However, a noticeable divergence between the training and validation curves emerges in later epochs, with the training loss continuing to decrease while the validation loss plateaus or exhibits mild fluctuations. This behavior is indicative of a moderate generalization gap, suggesting that while the model effectively fits the training data, its ability to generalize to unseen sequences is somewhat constrained. From a statistical learning perspective, this pattern is consistent with limited dataset size and diversity, where the model begins to capture dataset-specific noise or subtle biases. Biologically, this may reflect insufficient coverage of the diverse sequence–structure motifs underlying allergenicity, leading to incomplete representation of the functional landscape.

In contrast, the augmented dataset (Figure S1B) demonstrates markedly improved optimization stability. Both training and validation losses decrease smoothly and remain closely aligned throughout the training process, with minimal oscillations or divergence. This behavior indicates efficient convergence and suggests that augmentation enhances the effective sampling of the peptide sequence manifold, thereby regularizing the learning process. The close agreement between training and validation losses may initially suggest strong generalization; however, when interpreted in conjunction with increased motif-level and semantic similarity

observed in the augmented dataset, it also reflects a denser and more homogeneous embedding space. In such a scenario, the model benefits from repeated exposure to similar sequence patterns, which can simplify the optimization landscape and reduce variance in gradient updates, but may also mask subtle overfitting effects if redundancy is not carefully controlled.

The augmented dataset with test dataset splitting before augmentation (Figure S1C) provides the most robust and interpretable training dynamics. Here, both training and validation losses again decrease smoothly and converge to low values, but importantly, this occurs under conditions where motif-level and semantic leakage have been explicitly minimized. The sustained alignment between the two curves, combined with the absence of late-epoch divergence, indicates that the model achieves stable generalization without relying on redundant or overlapping information between training and validation sets. From a geometric standpoint, this reflects learning within a well-structured embedding manifold where class-specific regions are sufficiently populated yet remain independent across dataset partitions. The absence of significant oscillations further suggests that the combined use of adaptive learning rate scheduling, gradient clipping, and regularization mechanisms (dropout, label smoothing, and layer normalization) effectively stabilizes the optimization process, even in a high-capacity multimodal architecture.

Overall, the progression from Figure S1A to S1C illustrates how dataset augmentation and, critically, leakage-aware splitting reshape the optimization landscape of the model. While augmentation alone improves convergence and reduces apparent generalization error, only the controlled splitting scenario ensures that these improvements reflect genuine learning rather than redundancy-induced bias. The consistency of low and stable loss values across training and validation in the final configuration provides strong evidence that the model successfully captures transferable sequence–structure representations of allergenicity, leveraging both the evolutionary priors encoded in ESM embeddings and the fine-grained temporal modeling capabilities of the TCN–LSTM architecture.

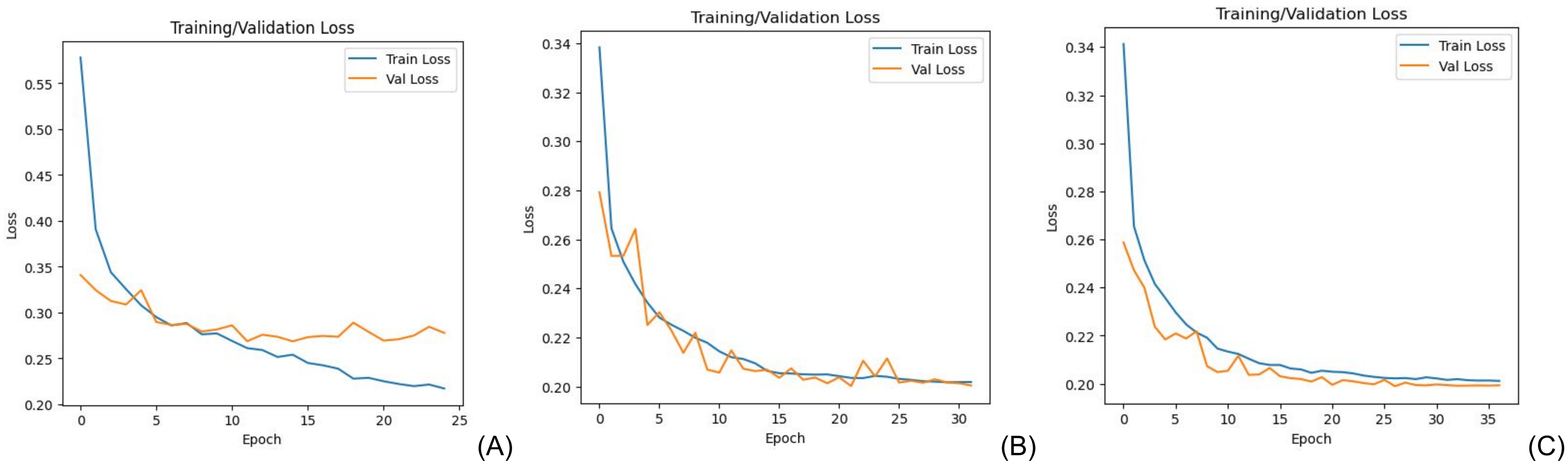

Figure S1. Training (blue line) and validation (orange line) losses for balanced augmented dataset with 35 epochs during optimization of the deep learning model (TCN + LSTM) to respectively classify allergen peptides using an Evolutionary Scale Modeling (ESM) language model (esm2_t6_8M_UR50D) to capture both global semantic and local structural features. (A) original dataset (2236 sequences for training and 559 sequences for validation), (B) augmented dataset (32000 sequences for training and 8000 sequences for validation), and (C) augmented original dataset with splitting (32000 sequences for training and 8000 sequences for validation).

Figures S2–S4 present confusion matrices for both training (left panels) and validation (right panels) datasets across the three experimental configurations—original dataset, augmented dataset, and augmented dataset with controlled splitting—after 35 epochs of optimization of the multimodal ESM–TCN–LSTM model. These matrices provide a granular decomposition of classification performance into true positives (TP), true negatives (TN), false positives (FP), and false negatives (FN), thereby enabling a detailed assessment of model sensitivity, specificity, and error distribution in the context of allergen peptide classification.

In the original dataset (Figure S2), the training confusion matrix exhibits a high number of correctly classified peptides (TP and TN), reflecting the model's strong capacity to learn discriminative features from limited data. However, the validation matrix reveals a more nuanced pattern, with a noticeable presence of false negatives and, to a lesser extent, false positives. This imbalance is consistent with the reduced recall and F1-score observed in the performance metrics, indicating that the model is more conservative in predicting allergenicity when exposed to unseen sequences. From a biological perspective, this suggests that certain allergenic motifs or sequence contexts present in the validation set are underrepresented in the training data, leading to missed detections. Geometrically, this corresponds to partial overlap between allergen and non-allergen regions in the embedding space, where decision boundaries are not fully optimized due to limited sampling of the sequence manifold.

The augmented dataset (Figure S3) shows a dramatic improvement in both training and validation confusion matrices, with near-perfect classification characterized by minimal FP and FN counts. The near-complete dominance of TP and TN entries indicates that the model effectively separates allergen and non-allergen peptides after augmentation. This reflects the increased density and coverage of the sequence space, which enables the model to learn smoother and more robust decision boundaries. However, this near-perfect performance must be interpreted cautiously, as augmentation introduces repeated or highly similar motifs that can reduce the effective complexity of the classification task. From a statistical standpoint, the reduction of both FP and FN errors suggests a

highly confident classifier, but this confidence may partially arise from increased redundancy and local similarity within the dataset, as supported by the elevated *k-mer* and semantic overlap observed in the leakage analysis.

In the augmented dataset with test dataset splitting before augmentation augmented dataset with test dataset splitting before augmentation (Figure S4), the confusion matrices retain near-perfect classification performance for both training and validation sets, with negligible FP and FN counts. Crucially, this high level of accuracy is achieved under conditions where motif-level and semantic leakage have been explicitly minimized, as demonstrated in the leakage analysis. The balanced distribution of TP and TN predictions, coupled with the absence of systematic error patterns, indicates that the model generalizes effectively to independent data. From a machine learning perspective, this confirms that the classifier is not relying on memorization of redundant motifs but is instead capturing underlying sequence–structure relationships that define allergenicity. Biologically, this suggests that allergenic peptides occupy a distinct yet internally diverse region of sequence space that can be robustly identified through multimodal representation learning.

The progression from Figure S2 to Figure S4 illustrates the critical role of dataset design in shaping model performance. While the original dataset reveals limitations in generalization due to restricted diversity, augmentation enhances classification accuracy by expanding the sequence manifold. Importantly, the controlled splitting strategy demonstrates that near-perfect classification can be maintained even in the absence of redundancy-driven leakage, thereby providing strong evidence that the model's predictive performance is grounded in biologically meaningful features rather than artifacts of data construction.

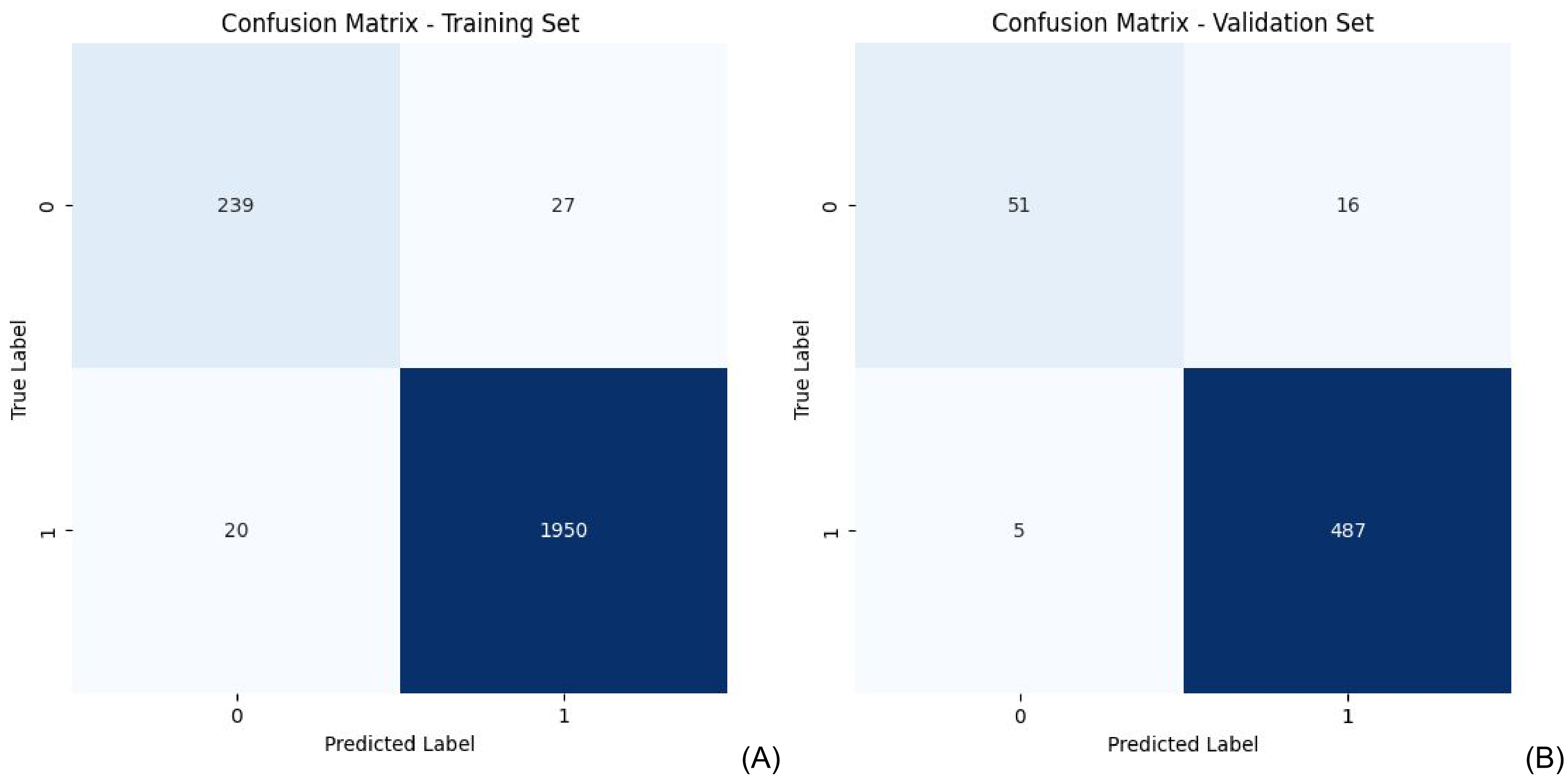

Figure S2. Confusion matrices of training (left panel, A) and validation (right panel, B) original dataset after 35 epoch optimizations of the deep learning model (TCN + LSTM) to respectively classify allergen peptides using an Evolutionary Scale Modeling (ESM) language model (esm2_t6_8M_UR50D) to capture both global semantic and local structural features.

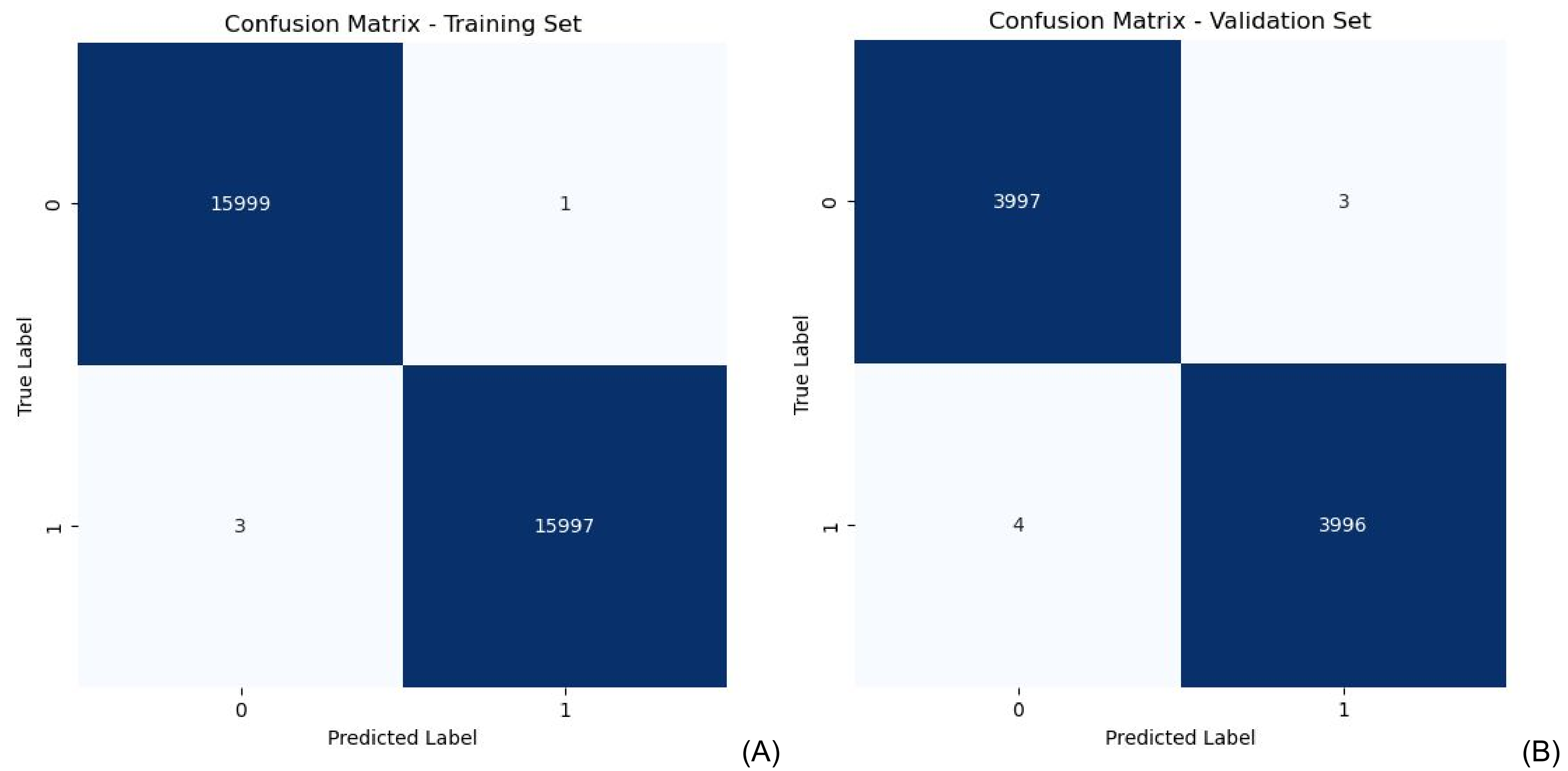

Figure S3. Confusion matrices of training (left panel, A) and validation (right panel, B) augmented datasets after 35 epoch optimizations of the deep learning model (TCN + LSTM) to respectively classify allergen peptides using an Evolutionary Scale Modeling (ESM) language model (esm2_t6_8M_UR50D) to capture both global semantic and local structural features.

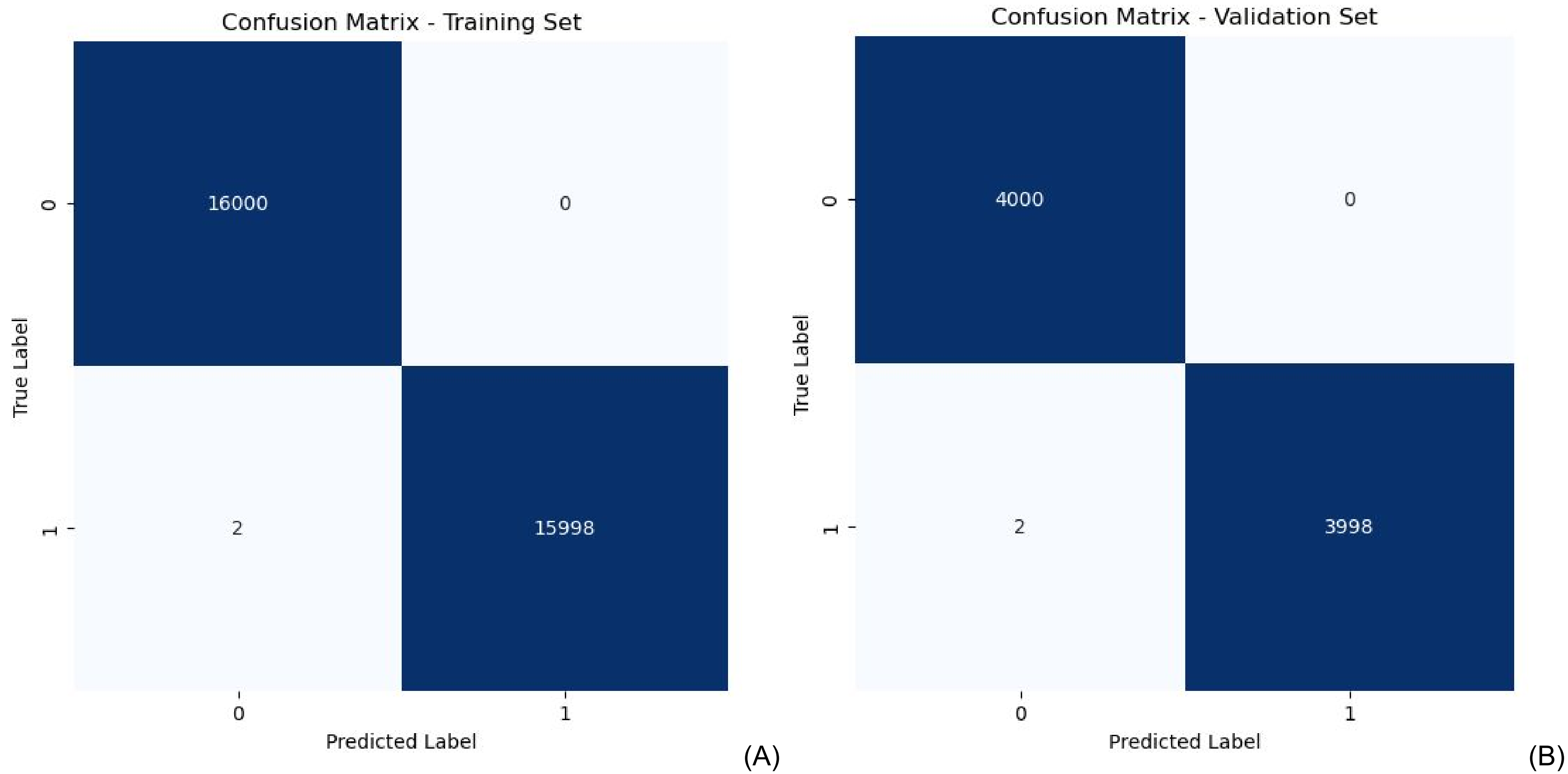

Figure S4. Confusion matrices of training (left panel, A) and validation (right panel, B) augmented original datasets with splitting after 35 epoch optimizations of the deep learning model (TCN + LSTM) to respectively classify allergen peptides using an Evolutionary Scale Modeling (ESM) language model (esm2_t6_8M_UR50D) to capture both global semantic and local structural features.

Figures S5 and S6 provide a geometric and manifold-level interpretation of the multimodal representation space learned by the integrated ESM–TCN–LSTM architecture, using two complementary nonlinear dimensionality reduction techniques: Uniform Manifold Approximation and Projection (UMAP) and t-Distributed Stochastic Neighbor Embedding (t-SNE). These methods project high-dimensional embeddings—encoding both global evolutionary semantics from the ESM2 model and local sequence-derived structural features—into a two-dimensional latent space while preserving neighborhood relationships. The resulting visualizations enable direct inspection of class separability, intra-class dispersion, and the global topology of allergen versus non-allergen peptide distributions across the three dataset configurations.

In the original dataset (Figures S5A and S6A), both UMAP and t-SNE projections reveal partially overlapping clusters corresponding to allergen and non-allergen peptides, indicating that the two classes share a substantial portion of the underlying biochemical and evolutionary feature space. This overlap is consistent with the relatively high inter-class similarity observed quantitatively and reflects the intrinsic complexity of allergenicity, where subtle sequence variations and context-dependent motifs govern immune recognition. From a geometric perspective, the embedding manifold appears moderately structured, with local neighborhoods forming around shared physicochemical properties, yet lacking a sharply defined global boundary between classes. Notably, t-SNE emphasizes local neighborhood preservation, revealing micro-clusters of peptides with similar motif compositions, whereas UMAP provides a more continuous representation of the global manifold, highlighting gradual transitions between allergenic and non-allergenic regions. Together, these projections suggest that, in the absence of augmentation, the model captures meaningful but only partially separable representations of allergenicity.

In the augmented dataset (Figures S5B and S6B), the embedding space undergoes a substantial transformation characterized by increased density and expansion of the manifold. Both UMAP and t-SNE visualizations show a clearer separation between allergen and non-allergen clusters, reflecting the reduced inter-class similarity and improved clustering metrics observed quantitatively. This

enhanced separability arises from the increased sampling of sequence space through augmentation, which effectively populates the latent manifold and reinforces class-specific regions. However, this densification is accompanied by a higher degree of local clustering, particularly visible in t-SNE as tightly packed micro-clusters, indicative of motif reuse and redundancy introduced by augmentation. From a representation learning standpoint, this results in smoother decision boundaries and facilitates classification, but it also raises the possibility that local neighborhoods may become overly homogeneous, potentially inflating model performance if not properly controlled.

The augmented dataset with test dataset splitting before augmentation (Figures S5C and S6C) provides the most informative representation of the intrinsic structure of allergenicity in the learned embedding space. In this configuration, UMAP and t-SNE both reveal well-separated yet internally heterogeneous clusters for allergen and non-allergen peptides. The separation between classes is maintained, consistent with the low inter-class similarity and high clustering quality metrics, while the reduction in motif-level redundancy ensures that clusters are not artificially compressed. Geometrically, this results in a manifold where allergen peptides occupy a distinct and coherent region, yet retain internal diversity, reflecting the multiplicity of sequence–structure patterns capable of eliciting immune responses. The agreement between UMAP and t-SNE projections is particularly significant, as it indicates that both global topology and local neighborhood structure consistently support class separation, reinforcing the robustness of the learned representations.

From a broader perspective, Figures S5 and S6 demonstrate that the multimodal embedding framework successfully captures the hierarchical organization of peptide sequence space, where allergenicity emerges as a structured but heterogeneous region within a continuous biochemical manifold. The progression from overlapping clusters in the original dataset to well-separated and biologically meaningful clusters in the augmented dataset with test dataset splitting before augmentation highlights the critical role of both data augmentation and leakage-aware splitting in shaping the geometry of the embedding space. Importantly, the consistency between

UMAP and t-SNE projections confirms that the observed separability is not an artifact of a specific dimensionality reduction method but reflects genuine structure in the high-dimensional representation learned by the model.

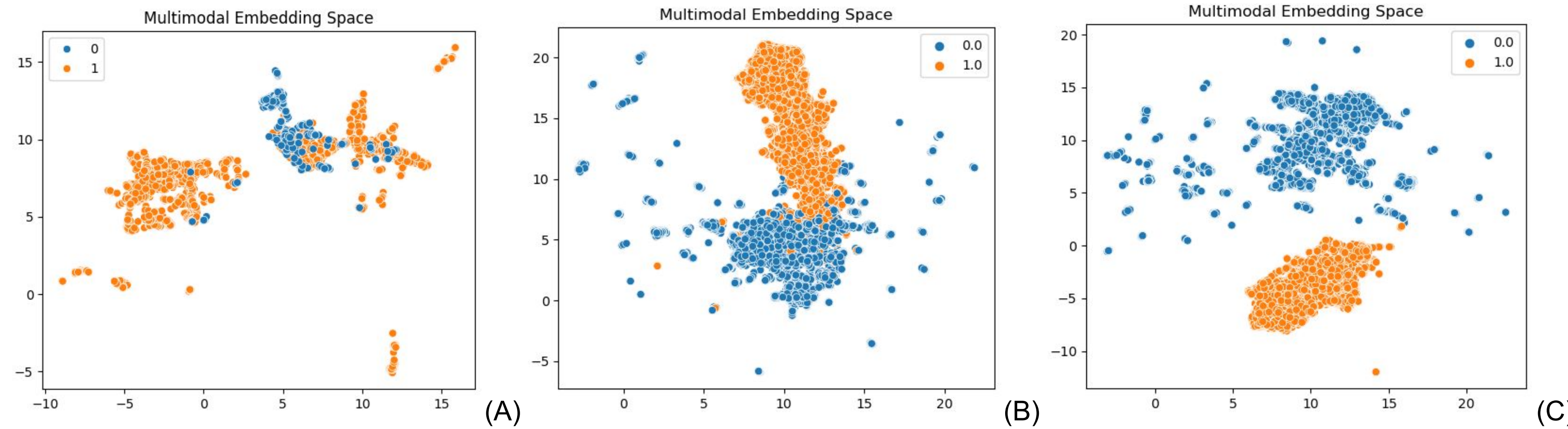

Figure S5. Multimodal embedding space for the (A) original dataset (2236 sequences for training and 559 sequences for validation), (B) augmented dataset (32000 sequences for training and 8000 sequences for validation), and (C) augmented original dataset with splitting (32000 sequences for training and 8000 sequences for validation) using the Uniform Manifold Approximation (UMAP) technique.

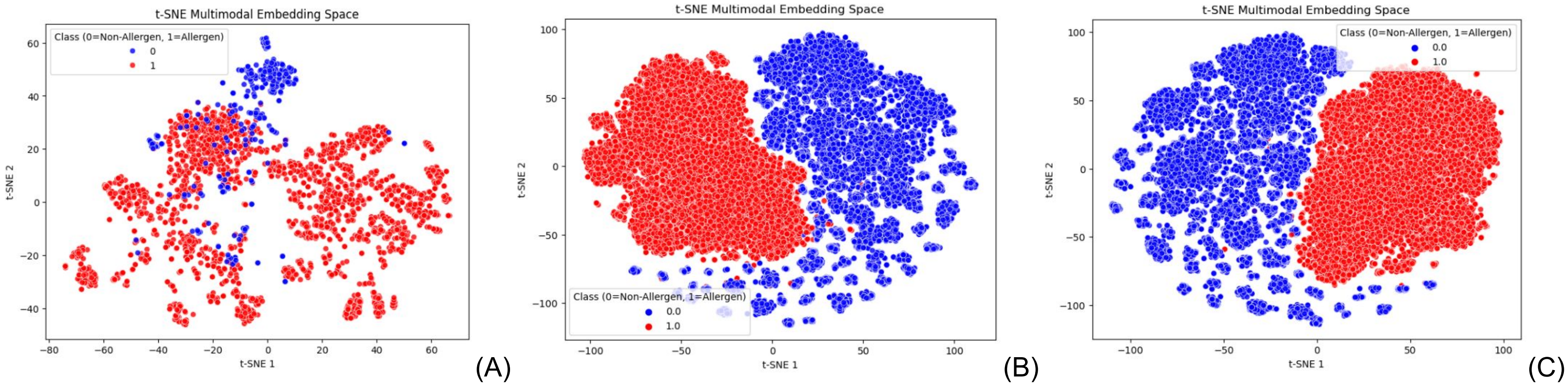

Figure S6. Multimodal embedding space for the (A) original dataset (2236 sequences for training and 559 sequences for validation), (B) augmented dataset (32000 sequences for training and 8000 sequences for validation), and (C) augmented original dataset with splitting (32000 sequences for training and 8000 sequences for validation) using the t-Distributed Stochastic Neighbor Embedding (t-SNE) technique.
